# Integrative metabolomic and transcriptomic analyses reveal flavonoid biosynthesis pathway in *Eupatorium lindleyanum*

**DOI:** 10.1038/s41598-025-27287-0

**Published:** 2025-12-04

**Authors:** Yingzhe Wang, Jinghan Wu, Min Zhou, Ximeng Yang, Yuan Niu, Kun Guo

**Affiliations:** 1 Jiangsu Food and Pharmaceutical Science College, Huaian, 223003 China; 2 JiLin Agricultural Science and Technology College, Jilin, 132109 China; 3 Lanzhou Agro-Technical Research and Popularization Center, Lanzhou, 730000 China

**Keywords:** Eupatorium lindleyanum, Flavonoids, Metabolomic, Transcriptomic, Secondary metabolism, Transcriptomics

## Abstract

*Eupatorium lindleyanum*, a medicinal plant from the Asteraceae family, is renowned for its diverse bioactive compounds, particularly flavonoids, which contribute to its various pharmacological activities. However, the biosynthetic pathway and regulatory mechanisms underlying flavonoid production in *Eupatorium lindleyanum* remain largely unexplored. In this study, an integrated metabolomic and transcriptomic approach was employed to investigate flavonoid biosynthesis in *Eupatorium lindleyanum*. Samples from four different tissues (roots, stems, leaves, and flowers) were analysed to identify variations in differentially expressed genes (DEGs) and differentially accumulated metabolites (DAMs). A total of 330 differentially accumulated flavonoid metabolites (DFMs) and 53,610 DEGs were identified. A total of 27 key structural genes involved in the flavonoid synthesis pathway, including *PAL*, *4CL*, *C3H*, *F3H*, *FLS*, and *ANS*, and others were found to be significantly activated in specific tissues. Additionally, 69 transcription factors (TFs) from five families, including *AP2/ERF*, *NAC*, *WRKY*, *MYB*, and *bHLH*, were identified as potentially involved in regulating flavonoid biosynthesis. The findings of this study offer crucial information on the genes and metabolites involved in flavonoid metabolism in *Eupatorium lindleyanum.* The identification of key genes and TFs, along with an understanding of their regulatory networks, can facilitate the development of new cultivars with increased flavonoid contents and improved medicinal value.

**Supplementary Information:**

The online version contains supplementary material available at 10.1038/s41598-025-27287-0.

## Introduction

*Eupatorium lindleyanum* is a perennial herbaceous plant in the Asteraceae family that is native to East Asia^1^. According to traditional Chinese medical theory, this plant is believed to resolve phlegm, relieve cough, and alleviate asthma^2^. The medicinal properties of *Eupatorium lindleyanum* were first investigated in the 1970s. It contains a variety of chemical constituents, including flavonoids, terpenes, and alkaloids^3^.Previous studies have identified several bioactive flavonoids, such as rutin, quercetin, eupafolin, jaceosidin, luteolin, hyperoside, isoquercitrin, kaempferol, caffeic acid, and eupatilin, in *Eupatorium lindleyanum*. These compounds have been shown to exhibit strong effects in protecting against lung injury, reducing blood lipid levels, combating tumors, reducing inflammation, and scavenging free radicals, supporting the traditional medicinal uses of the plant^4^. Given their significant medicinal potential, these bioactive flavonoids have been identified as key metabolic targets and candidates for future metabolic engineering.

However, the biosynthetic pathway of flavonoid in *Eupatorium lindleyanu*m has not been fully elucidated. While the general framework of the flavonoid biosynthetic pathway in plants has been essentially elucidated, recent studies in the Asteraceae family have revealed unique advancements and variations in this pathway^5^. For instance, the identification and characterization of flavone synthase II (*FNS II*) in *Chrysanthemum indicum* have shown its significant role in flavonoid accumulation and stress response^6^. Additionally, the discovery of specific glycosyltransferases involved in flavonoid glycoside biosynthesis in safflower (*Carthamus tinctorius*) has provided new insights into the regulation and modification of flavonoids^7^. Similarly, in *Echinacea angustifolia*, the identification of key enzymes (*F6H1*,* F6H2*,* CCoAOMT1/2*) responsible for the biosynthesis of the rare flavonol patuletin has enhanced our understanding of the unique flavonoid biosynthetic pathway in this medicinal plant and enabled the reconstruction of patuletin biosynthesis in *Nicotiana benthamiana* leaves^8^. These findings highlight the unique aspects of flavonoid biosynthesis in the Asteraceae family, which are not fully captured by the traditional view of a highly conserved pathway. In *Eupatorium lindleyanum*, none of the genes encoding the key enzymes in the flavonoid biosynthetic pathway have been fully characterized. This gap in knowledge extends to the regulation of this pathway in different tissues, which remains poorly understood. It is unclear whether tissue-specific regulation of flavonoid biosynthesis in *Eupatorium lindleyanum* is unique compared to other plants. Further research is needed to identify the specific regulatory mechanisms and tissue-specific expression patterns of these enzymes, which could provide insights into the unique medicinal properties of this plant. Given the significant medicinal potential of these bioactive flavonoids, elucidating their biosynthetic pathway in *Eupatorium lindleyanum* holds substantial application value. Firstly, it enables the optimization of bioactive flavonoid production through genetic engineering and metabolic regulation, enhancing the yield of specific compounds like quercetin and eupatilin. Secondly, this knowledge facilitates the breeding of *Eupatorium lindleyanum* varieties with higher concentrations of medicinal flavonoids, improving therapeutic efficacy. Thirdly, understanding this pathway can optimize cultivation practices to maximize bioactive compound production under different environmental conditions. In summary, elucidating this pathway supports the development of more effective herbal medicines, improved cultivation practices, and the discovery of new pharmaceuticals, thereby enhancing the overall medicinal value of *Eupatorium lindleyanum*.

Metabolomics and transcriptomics are powerful tools for studying the biosynthesis and regulation of secondary metabolites in plants. Metabolomics involves the comprehensive analysis of small molecules (metabolites) in biological samples, providing insights into metabolic profiles and biochemical pathway^9^. On the other hand, transcriptomics focuses on the analysis of gene expression patterns, revealing the regulatory networks and functional genes involved in metabolic processes. Integrating metabolomic and transcriptomic data allows for a holistic understanding of the molecular mechanisms underlying secondary metabolite production^10^. Specifically, RNA sequencing (RNA-seq) is used to detect differentially expressed genes (DEGs) under different conditions, whereas liquid chromatography‒tandem mass spectrometry (LC‒MS/MS) can be used to identify and quantify metabolites. Correlation analysis between DEGs and differentially accumulated metabolites (DAMs) helps to pinpoint genes that are significantly associated with specific metabolite changes. Pathway enrichment analysis maps these genes and metabolites to known biosynthetic pathways, revealing regulatory networks and key steps in the biosynthesis of secondary metabolites. This comprehensive strategy not only identifies critical genes and metabolites but also reveals the regulatory mechanisms governing their production, providing valuable insights into the complex metabolic networks underlying plant secondary metabolism^11^. For example, this approach has been successfully applied in various species, such as *Glycyrrhiza uralensis*^12^, *Ziziphi Spinosae*^13^, *Perilla Frutescens*^14^, and *Lilium davidii*^15^, to elucidate the biosynthetic pathway of flavonoid and other secondary metabolites.

In this study, we collected samples from four distinct tissues—roots, stems, leaves, and flowers—and performed comprehensive metabolite profiling and gene expression analysis. We then employed metabolomic and transcriptomic analyses to investigate the biosynthesis and regulation of flavonoids in *Eupatorium lindleyanum*. From the transcriptome analysis, we identified differentially expressed genes and transcription factors in the six pairs of comparisons. Additionally, metabolomic analysis revealed differentially accumulated metabolites across different tissues. Furthermore, we established a flavonoid biosynthetic regulatory network in *Eupatorium lindleyanum* via KEGG coenrichment and correlation analyses of DAMs and DEGs. These findings can be applied to genetic engineering programs and breeding initiatives aimed at developing new cultivars with increased flavonoid contents in *Eupatorium lindleyanum*. Moreover, the identification of key regulatory genes and transcription factors involved in flavonoid biosynthesis will provide potential targets for genetic modification to increase the medicinal value by increasing the flavonoid content.

## Materials and methods

### Plant materials

The experimental materials, *Eupatorium lindleyanum* plants, were collected from their natural habitat in Xuyi County, Jiangsu Province (32°55′37.831″N, 118°25′46.878″E), on 14 September 2024, a time chosen for sampling as it coincides with the peak growth period of the plant, during which the synthesis and accumulation of secondary metabolites, including flavonoids, reach relatively high levels^16^. Prior to collection, a scientific research permit was obtained from the Jiangsu Provincial Forestry Bureau and the Huaian Municipal Bureau of Natural Resources and Planning, ensuring compliance with the Regulations on the Protection of Wild Plants of the People’s Republic of China. Additionally, the climatic conditions in September are relatively stable, with suitable temperature and humidity, thereby minimizing the potential interference from environmental variability. The plants were in the full flowering stage at the time of collection, with well-developed roots, stems, leaves, and flowers. The selected plants were of similar age and size, ensuring physiological uniformity across the samples. The plants were healthy and free from visible signs of disease or pest infestation. The collection site was characterized by a temperate climate with an average daily temperature of 22℃ and relative humidity of 70%. The plants were exposed to natural sunlight and had access to adequate water supply from recent rainfall. The site was located in a relatively undisturbed area, away from industrial pollution and urban development. The plant materials used in this study were formally identified by Professor Meng Zhu from Jiangsu Food and Pharmaceutical Science College based on morphological characteristics described in Flora of China (http://www.efloras.org/florataxon.aspx?flora_id=2&taxon_id=200023938), and the detailed description of the plant tissues is shown in Additional Fig. 1. A voucher specimen (NAS00718179) has been deposited in the publicly accessible herbarium of Nanjing Botanical Garden, Chinese Academy of Sciences, and is available for examination upon request to the curator (bgs@cnbg.net). Four plant tissues—roots, stems, leaves, and flowers—were harvested from three individual plants. For each tissue type, three biological replicates were prepared by separately collecting samples from three distinct individual plants, ensuring that each replicate was independent and representative of the tissue type. Immediately after collection, the plant materials were thoroughly cleaned with ultrapure water, flash-frozen in liquid nitrogen, and stored at -80 °C for subsequent transcriptome sequencing and metabolomic analysis.

### Processing of plant samples and extraction of metabolites

The extraction method for metabolites was derived from previous studies with minor modifications^17^. The four tissues of *Eupatorium lindleyanum* were processed into powder via a ball mill (30 Hz, 1.5 min, Scientz-100 F, Ningbo, China) after lyophilization. Fifty milligrams of powder was weighed and dissolved in 1200µL of 70% methanol, which had been precooled to -20 °C. The use of 1200µL of 70% methanol for 50 mg of tissue ensures effective extraction of both polar and non-polar metabolites by balancing solubility and cell disruption, while also guaranteeing thorough tissue immersion to maximize metabolite release. The mixture was subsequently vortexed for 30 s, which was repeated every 30 min, for a total of 6 times. After centrifugation, the supernatant was collected and filtered through a microporous membrane (0.22 μm pore size) to remove impurities for subsequent UPLC‒MS/MS analysis^18^.

### UPLC‒MS/MS analysis conditions for metabolite profiling

The sample extracts were analysed via a UPLC‒MS/MS system (UPLC, ExionLC™ AD, https://sciex.com.cn/). The separation of metabolites was performed on an Agilent SB-C18 (1.8 μm, 2.1 mm * 100 mm) chromatographic column. Mobile phase A: pure water with 0.1% formic acid; mobile phase B: acetonitrile with 0.1% formic acid. Sample measurements were performed via a gradient program with the following conditions: 95% A and 5% B. Within 9 min, the gradient was linearly adjusted to 5% A and 95% B and maintained for 1 min. The composition was subsequently adjusted back to 95% A and 5% B within 1.1 min and held for an additional 2.9 min. The flow velocity was set at 0.35mL/min; the column oven temperature was maintained at 40 °C; and the injection volume was 2µL. The effluent was directed to an electrospray ionization (ESI)-triple quadrupole-linear ion trap (QTRAP)-MS detector.

The ESI source operation parameters were as follows: source temperature: 500 °C; ion spray voltage (IS), 5500 V (positive ion mode)/-4500 V (negative ion mode); ion source gas I (GSI), gas II (GSII), and curtain gas (CUR) were set at 50 psi, 60 psi, and 25 psi, respectively; and collision-activated dissociation (CAD) was set to high. QQQ scans were acquired in MRM mode with the collision gas (nitrogen) set to medium.

### Qualitative and quantitative analysis of metabolites

The qualitative analysis of metabolites was conducted by comparing secondary fragment ions with the MetWare database (MetWare Biotechnology Co. Ltd., Wuhan, China). The quantitative analysis of metabolites was performed via triple quadrupole mass spectrometry in MRM mode. All mass spectrometry data were converted via Analyst 1.6.3 software^19^. Unsupervised principal component analysis (PCA) was performed via the statistics function prcomp within R software (version 3.5.1). Variable importance in projection (VIP) values were extracted from the OPLS-DA results via the R package MetaboAnalyst 6.0 (https://metaboanalyst.ca). Differentially accumulated metabolites (DAMs) were identified among the six pairwise comparisons using combined criteria: variable importance in projection (VIP) > 1, |log2fold change|≥1, and false discovery rate (FDR) < 0.05 (Benjamini-Hochberg correction). The differentially accumulated metabolites were subsequently annotated via the KEGG database (http://www.kegg.jp/kegg/compound/)^20^.

### RNA extraction and transcriptome sequencing

The plant samples were processed into powder via liquid nitrogen, and RNA was subsequently isolated using Total RNA Kit (TianGen, Beijing, China). After successful extraction, the RNA was dissolved by adding 50µL of DEPC-treated water. The total RNA was subsequently identified and quantified via a Qubit fluorescence quantifier (Thermo Fisher Scientific, Waltham, MA, USA) and a Qsep400 high-throughput biofragment analyser (Phonton Biotech, Taiwan, China). The mRNAs with polyA tails were enriched with oligo (dT) magnetic beads and then reverse transcribed into cDNAs via the PrimeScript™ RT reagent Kit (TaKaRa, Dalian, China). The cDNA library was constructed by connecting sequencing adapters at both ends. After the initial library was constructed, a Qubit fluorescence quantifier was used for concentration detection, followed by a Qsep400 high-throughput biofragment analyser for fragment size detection. After passing the library check, which included both concentration and size verification, the libraries were sequenced through the Illumina HiSeq platform (Illumina, USA)^21^.

### Data analysis of transcriptome sequences

To obtain clean reads, the raw reads were filtered by using fastp software v0.23.2.Transcriptome assembly of the clean reads was performed via Trinity v2.15.1 using a merged assembly approach to construct a comprehensive transcriptome library, which is particularly suitable for multiple samples from the same species. This method ensures a more complete representation of transcripts, facilitating subsequent expression quantification and differential expression analysis. The assembled transcripts were then clustered and de-redundant using Corset v1.09. Transcript sequences after de-redundancy were aligned against the Kyoto Encyclopedia of Genes and Genomes (KEGG), Non-Redundant (NR), Swiss-Prot, Gene Ontology (GO), euKaryotic Orthologous Groups (KOG), and Translated EMBL databases via DIAMOND software v2.1.6 to obtain the annotation information of the transcripts from these six major databases. The expression levels of the transcripts were calculated via RSEM software v1.3.3, and the FPKM value of each transcript was subsequently calculated according to the transcript length. The differential expression between two plant tissues was calculated via DESeq2 software v1.47.1, and the false discovery rate (FDR) control was applied to adjust the threshold of the *P*-value. Genes with absolute log2fold change ≥ 1 and FDR < 0.05 were defined as DEGs. Transcription factor prediction was performed via iTAK v2.0.2^22–27^.

### Combined metabolome and transcriptome analysis

To elucidate the underlying mechanisms of flavonoid biosynthesis in *Eupatorium lindleyanum*, we performed an integrated analysis of the transcriptome and metabolome. First, we identified DAMs and DEGs on the basis of a fold change of > 2. The KEGG pathways that were coenriched with these DAMs and DEGs were then selected for further analysis. Specifically, DEGs and DAMs that were coenriched in five flavonoid biosynthesis pathways were selected across the six pairs of comparisons. These pathways include phenylpropanoid biosynthesis (ko00940), flavonoid biosynthesis (ko00941), anthocyanin biosynthesis (ko00942), isoflavonoid biosynthesis (ko00943), and flavone and flavonol biosynthesis (ko00944). For the combined analysis, log2-transformed data of genes and metabolites were utilized to conduct correlation analysis. Genes and metabolites with Pearson’s correlation coefficients greater than 0.80 (absolute value) and *P <* 0.05 were subsequently selected^28^. These selected genes and metabolites were then visualized in a correlation network diagram via Cytoscape (version 3.10.3, https://cytoscape.org). On the basis of the expression patterns of these selected genes and metabolites, we reconstructed the biosynthetic pathway of flavonoids in *Eupatorium lindleyanum*. Key enzymes and metabolites involved in the pathway were identified, and their interactions were elucidated.

### Quantitative real-time PCR validation of candidate genes

The extracted RNA was reverse transcribed into cDNA via a MonScript™ RTIII reagent kit (Monad Biotech, Suzhou, China). The primers for 10 candidate genes and a internal reference gene were designed using Primer Premier 5.0 software (http://www.premierbiosoft.com/primerdesign/index.html), and the amplification efficiency for each primer was calculated, as detailed in Additional Table 1. The qRT‒PCR system was performed via the QuantiNova SYBR Green PCR Kit (Qiagen Biotech, Hilden, Germany) and detected via the ABI QuantStudio 6 (Thermo Fisher, USA) according to the manufacturer’s instructions. Each sample was tested in triplicate. The β-actin gene was used as the interal reference gene, and the relative expression levels of the genes were calculated using the 2^−∆∆Ct^ method^29^. The internal reference gene β-actin was subjected to stability assessment using three software tools: geNorm version 3.5, NormFinder version 0.953, and BestKeeper version 1.0. The M value calculated by geNorm was 0.527, the stability value determined by NormFinder was 0.082, and the standard deviation value computed by BestKeeper was 0.453. The analysis results indicate that β-actin exhibits good expression stability and can be used as a reference gene.

## Results

### Analysis of metabolome profiling in different tissues of *Eupatorium lindleyanum*

**Fig. 1:**
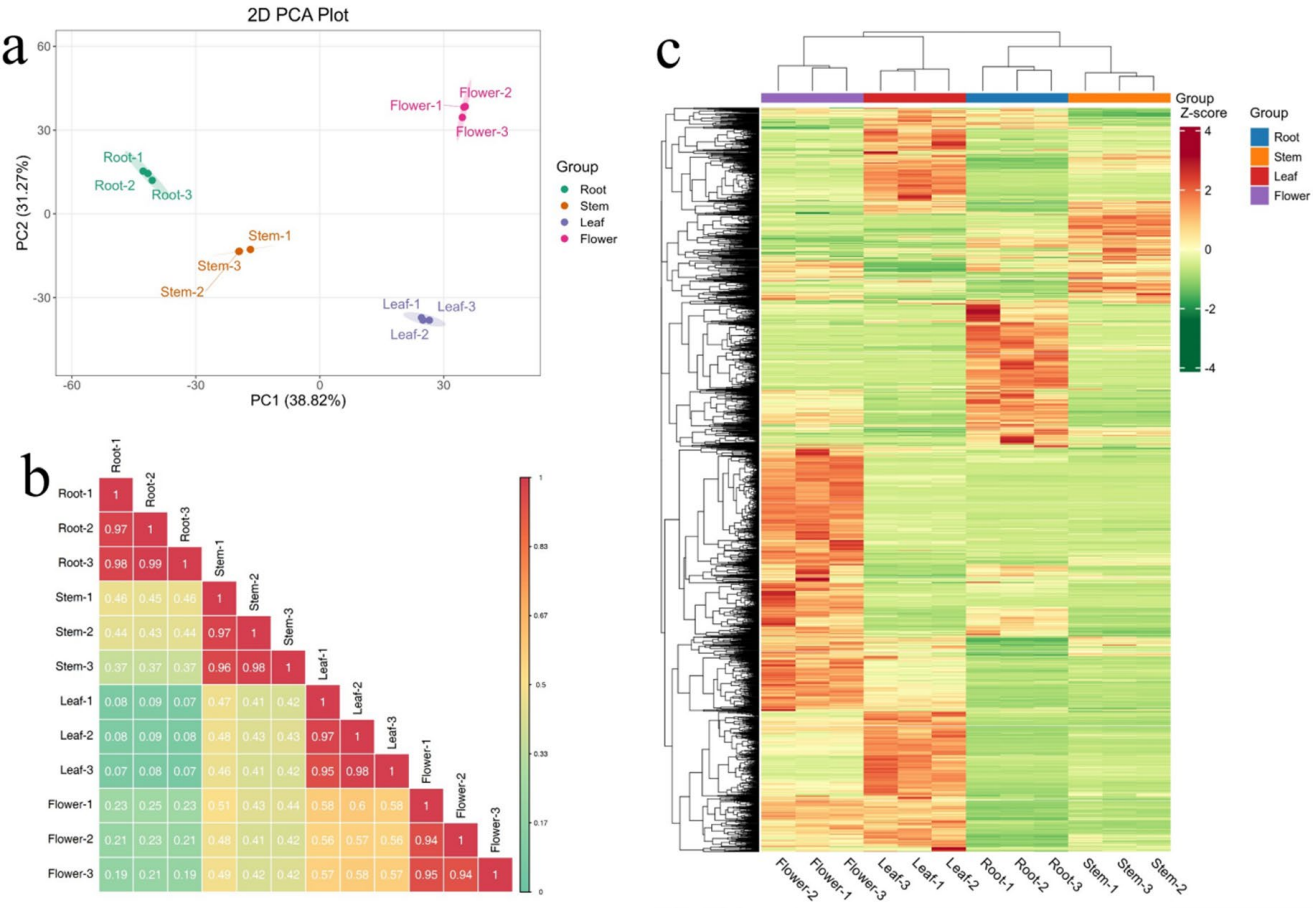
Metabolomic profiling of *Eupatorium lindleyanum* and its biological replicates. (**a**) Principal component analysis of different tissues. (**b**) Pearson correlation analysis between 12 samples. (**c**) Hierarchical clustering analysis of 2770 metabolites in 12 samples: color intensity reflects relative abundance.

A total of 2770 metabolites were detected in four tissues of *Eupatorium lindleyanum* using UPLC-MS/MS. These metabolites were categorized into the following groups: 238 alkaloids, 186 amino acids and derivatives; 338 flavonoids; 169 lignans and coumarins; 258 lipids; 64 nucleotides and derivatives; 123 organic acids; 256 phenolic acids; 30 quinones; 21 steroids; 4 tannins; 600 terpenoids; and 483 other metabolites (See Additional Fig. 2 and Additional Table 2 for details). Principal component analysis (PCA) of metabolites from different tissues of *Eupatorium lindleyanum* are presented in Fig. 1a. The analysis demonstrated that biological replicate samples from roots, stems, leaves, and flowers were distinctly clustered, with each tissue type forming a clear and well-separated group. This pattern emphasized the distinct tissue-specific biological signatures among the four tissues. The results of the correlation analysis also confirmed this pattern. As shown in Fig. 1b, there was a high correlation (typically above 0.9) between the biological replicates of each tissue type. In contrast, the correlation between tissues from roots, stems, leaves, and flowers was relatively weak, indicating distinct metabolic profiles for each tissue. Hierarchical clustering analysis (HCA) of 2770 metabolites further supported the unique metabolic profiles (as shown in Fig. 1c). This analysis revealed distinct differences in metabolite abundance among the various tissues of *Eupatorium lindleyanum*, highlighting the unique metabolic profiles associated with each tissue type.

### Differentially accumulated flavonoid metabolites in four tissues of *Eupatorium lindleyanum*

**Fig. 2:**
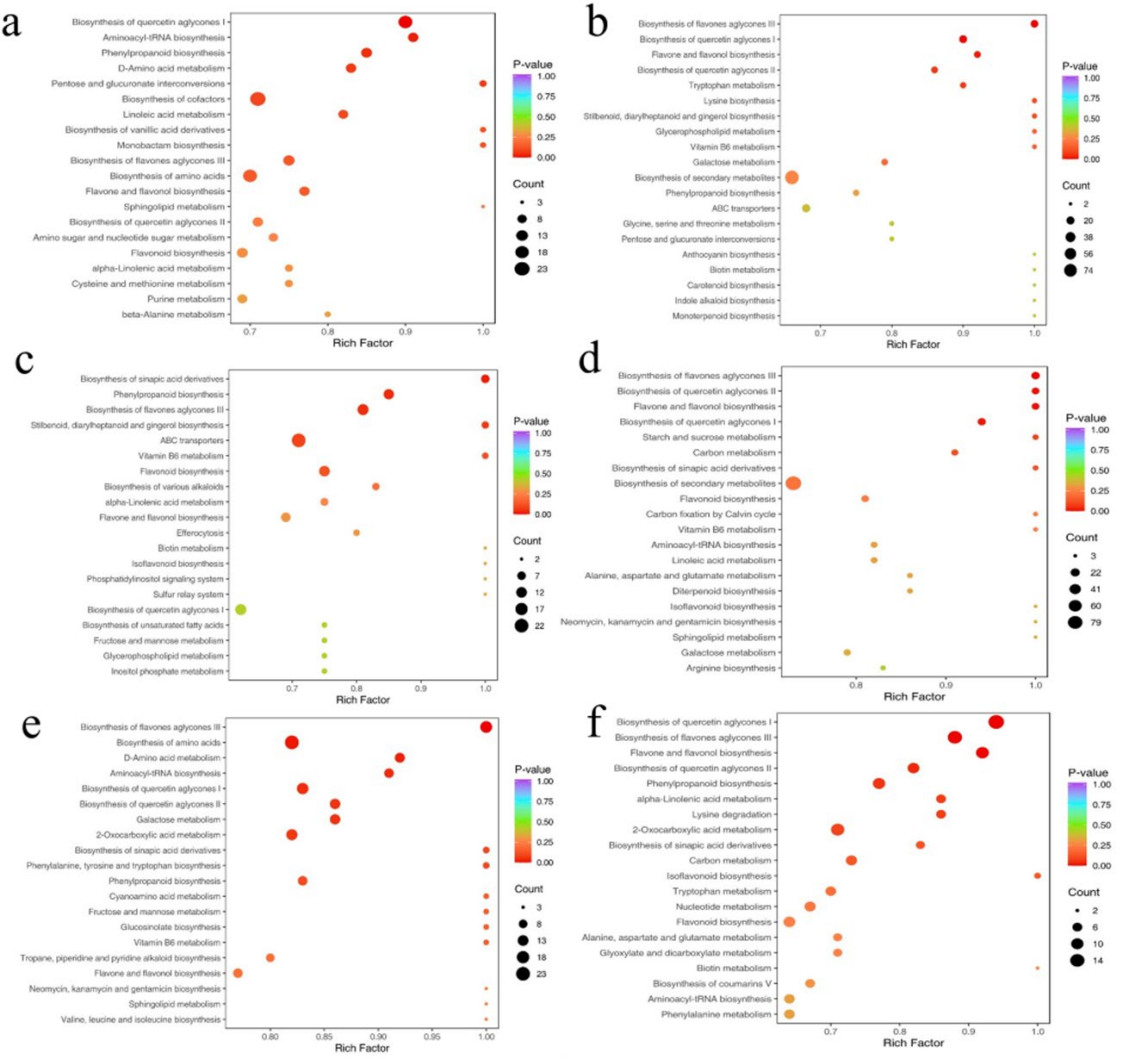
KEGG enrichment analysis of the DAMs. The horizontal axis represents the Rich Factor corresponding to each pathway. The vertical axis represents the pathway name. The color of the dots indicates the *P*-value, representing the statistical significance of the pathway enrichment. The size of the dots corresponds to the number of DAMs. The top 20 pathways with the lowest *P*-values are displayed. (**a**) Flower vs. leaf (**b**) flower vs. root (**c**) flower vs. stem (**d**) leaf vs. root (**e**) leaf vs. stem (**f**) stem vs. root. (Enrichment results were obtained from the Kyoto Encyclopedia of Genes and Genomes database and is reproduced with permission, www.kegg.jp/kegg/kegg1.html)

**Fig. 3:**
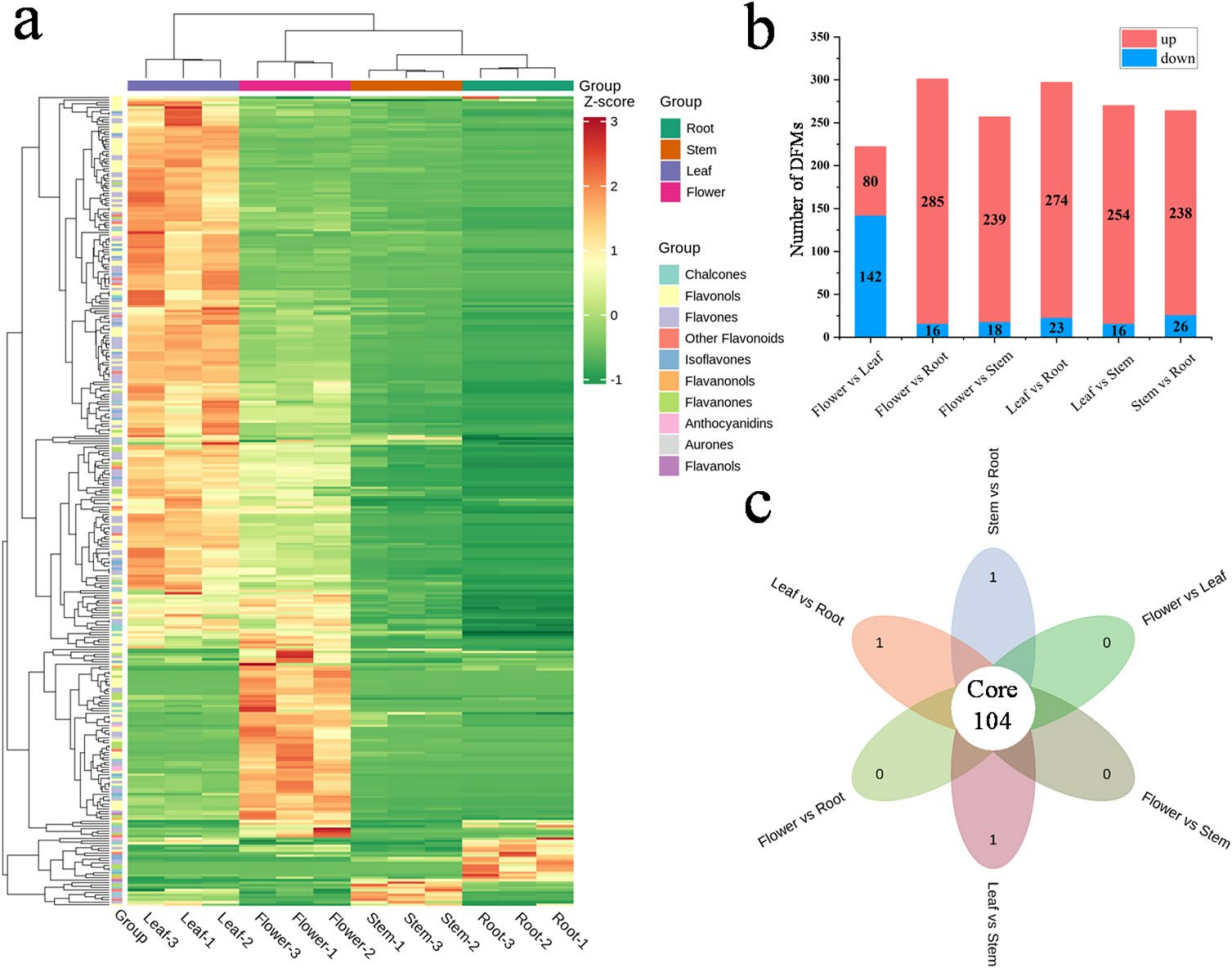
Profiles of DFMs for four tissues of *Eupatorium lindleyanum.* (**a**) Hierarchical clustering heatmap of the relative content of 330 DFMs. The content was color coded and normalized by the Z score. (**b**) Number of DFMs in the six pairs of comparisons. (**c**) Venn diagram of the six pairs of comparisons.

A total of 1860, 1700, 1578, 1694, 1899, and 1591 DAMs were filtered in the comparisons of flower vs. root, flower vs. stem, flower vs. leaf, stem vs. root, leaf vs. root, and leaf vs. stem, respectively. To elucidate the primary biosynthetic pathway associated with DAMs in *Eupatorium lindleyanum*, we performed KEGG pathway enrichment analysis. Across the six pairwise comparisons, a non-redundant set of 13 DAMs was mapped to and enriched in phenylpropanoid biosynthesis (ko00940), 15 DAMs in flavonoid biosynthesis (ko00941), 2 DAMs in anthocyanin biosynthesis (ko00942), 3 DAMs in isoflavonoid biosynthesis (ko00943), and 13 DAMs in flavone and flavonol biosynthesis (ko00944) (Fig. 2). The differentially accumulated flavonoid metabolites (DFMs) were subsequently screened among the DAMs in each pair of comparison. A total of 330 DFMs were identified across all the paired comparisons (See Additional Table 3). Flavonoids include mainly anthocyanidins, aurones, chalcones, flavanols, flavanones, flavanonols, flavones, flavonols, isoflavones, and other flavonoids (See Additional Fig. 3). The number of DFMs in each of the six pairs of comparisons is shown in Fig. 3b. Notably, the flower vs. root group presented the greatest number of DFMs (301), with 285 upregulated and 16 downregulated. In contrast, the flower vs. leaf group presented the lowest number of DFMs (222), with 80 upregulated and 142 downregulated DFMs. Other paired comparisons presented intermediate numbers of DFMs: flower vs. stem (257 DFMs, 239 upregulated and 18 downregulated), leaf vs. root (297 DFMs, 274 upregulated and 23 downregulated), leaf vs. stem (270 DFMs, 254 upregulated and 16 downregulated), and stem vs. root (264 DFMs, 254 upregulated and 10 downregulated). According to the Venn diagram (Fig. 3c), 104 DFMs were shared among all six pairs of comparisons. The hierarchical clustering heatmap revealed significant tissue-specific differences in the relative content of DFMs among various tissues of *Eupatorium lindleyanum* (Fig. 3a). For example, the stem contained very low levels of isoflavones and anthocyanins, and the root had almost no aurones. In contrast, the leaf was rich in flavonols and flavones.

### Comprehensive transcriptome analysis of *Eupatorium lindleyanum* in four distinct tissues

**Fig. 4:**
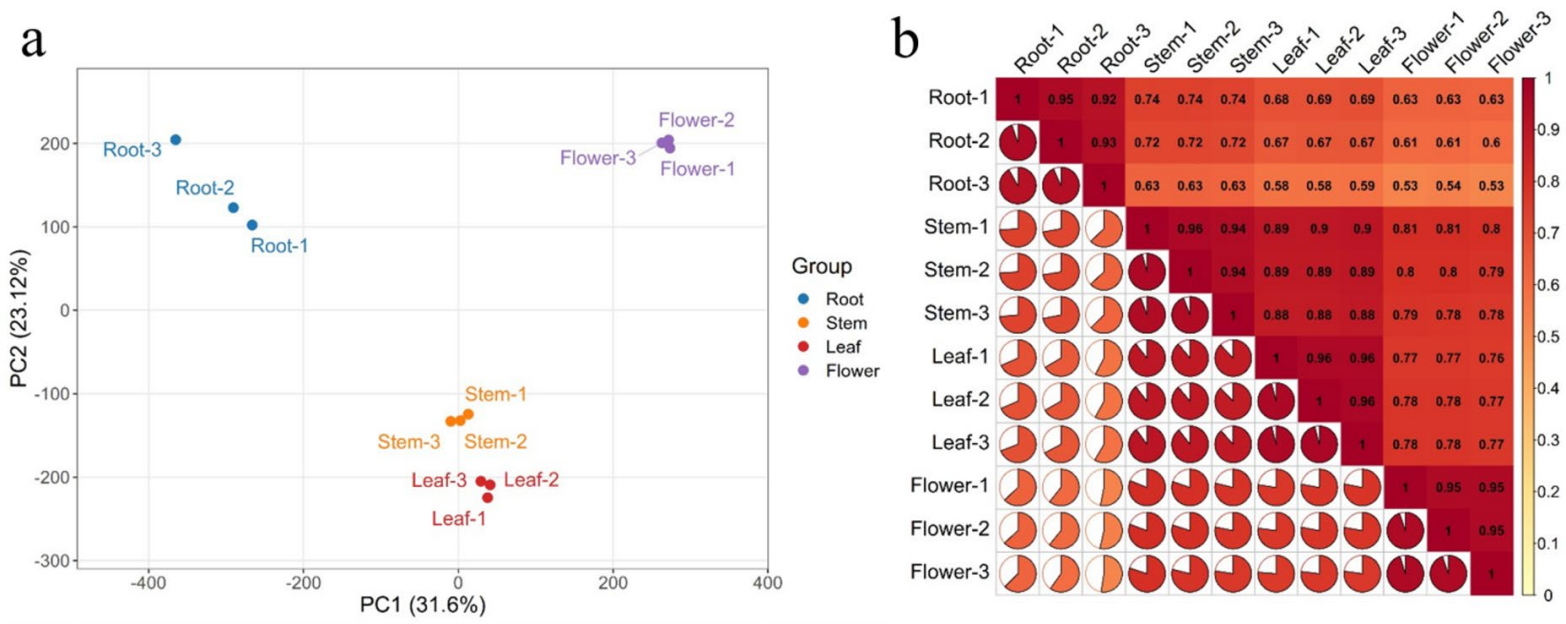
Principal component analysis (**a**) and correlation analysis (**b**) of transcriptome data from four tissues of *Eupatorium lindleyanum* (root, stem, leaf, and flower) and their replicates.

Transcriptome sequencing was performed on different tissues of *Eupatorium lindleyanum*. A total of 12 cDNA libraries were generated from four distinct tissues, each with three biological replicates. All the libraries were subsequently sequenced, yielding 47,983,328 to 69,716,676 raw reads. Next, 47,182,646 to 66,154,460 clean reads (a total of 99.43 Gb) were obtained by filtering the raw reads. The quality control parameters for the transcriptome data of the 12 samples were a Q20 ≥ 98.38% and a Q30 ≥ 95.35%. The GC content of each library ranged from 42.79% to 44.1% (Table 1). In summary, the quality of the transcriptome data was high, indicating successful sequencing and filtering processes. A total of 147,743 unigenes were obtained through transcript splicing and assembly. The unigenes were compared against multiple databases, resulting in the following annotations: 70,028 genes in KEGG, 90,760 in Nr, 65,385 in SwissProt, 91,058 in TrEMBL, 60,040 in KOG, and 80,518 in GO (See Additional Table 4). All unigenes were compared to the database, with 66.54% being annotated in at least one database. PCA and correlation analyses were performed to assess the repeatability within samples via fragments per kilobase of exon model per million mapped fragments (FPKM). The PCA results indicated that PC1 and PC2 accounted for 31.6% and 23.12% of the variability, respectively, with good separation between sample groups and high repeatability within groups (Fig. 4a). The correlation analysis results showed good repeatability within each group. Moreover, strong correlations in gene expression were detected between stems and leaves, a finding consistent with the pattern observed in the principal component analysis (Fig. 4b).

**Table 1:** Transcriptome data quality control parameters for 12 samples.

Sample	Group	Raw reads	Clean reads	Clean base (G)	Error rate (%)	Q20 (%)	Q30 (%)	GC content (%)
Flower-1	Flower	59,877,152	58,863,280	8.83	0.01	98.43	95.45	43.53
Flower-2	Flower	60,192,250	58,972,190	8.85	0.01	98.47	95.56	43.79
Flower-3	Flower	69,716,676	66,154,460	9.92	0.01	99.27	97.61	44.06
Leaf-1	Leaf	52,365,536	51,403,656	7.71	0.01	98.44	95.5	43.09
Leaf-2	Leaf	55,833,204	54,819,118	8.22	0.01	98.52	95.69	43.26
Leaf-3	Leaf	60,398,224	59,302,044	8.9	0.01	98.47	95.54	43.34
Root-1	Root	57,780,310	55,024,994	8.25	0.01	98.38	95.35	44.1
Root-2	Root	52,871,448	51,911,994	7.79	0.01	98.46	95.56	43.37
Root-3	Root	47,983,328	47,182,646	7.08	0.01	98.51	95.68	42.79
Stem-1	Stem	57,708,702	56,537,092	8.48	0.01	98.46	95.58	43.41
Stem-2	Stem	49,183,394	48,420,402	7.26	0.01	98.48	95.6	43.51
Stem-3	Stem	55,158,270	54,246,392	8.14	0.01	98.43	95.5	43.2

### Identification and functional enrichment analysis of DEGs

**Fig. 5:**
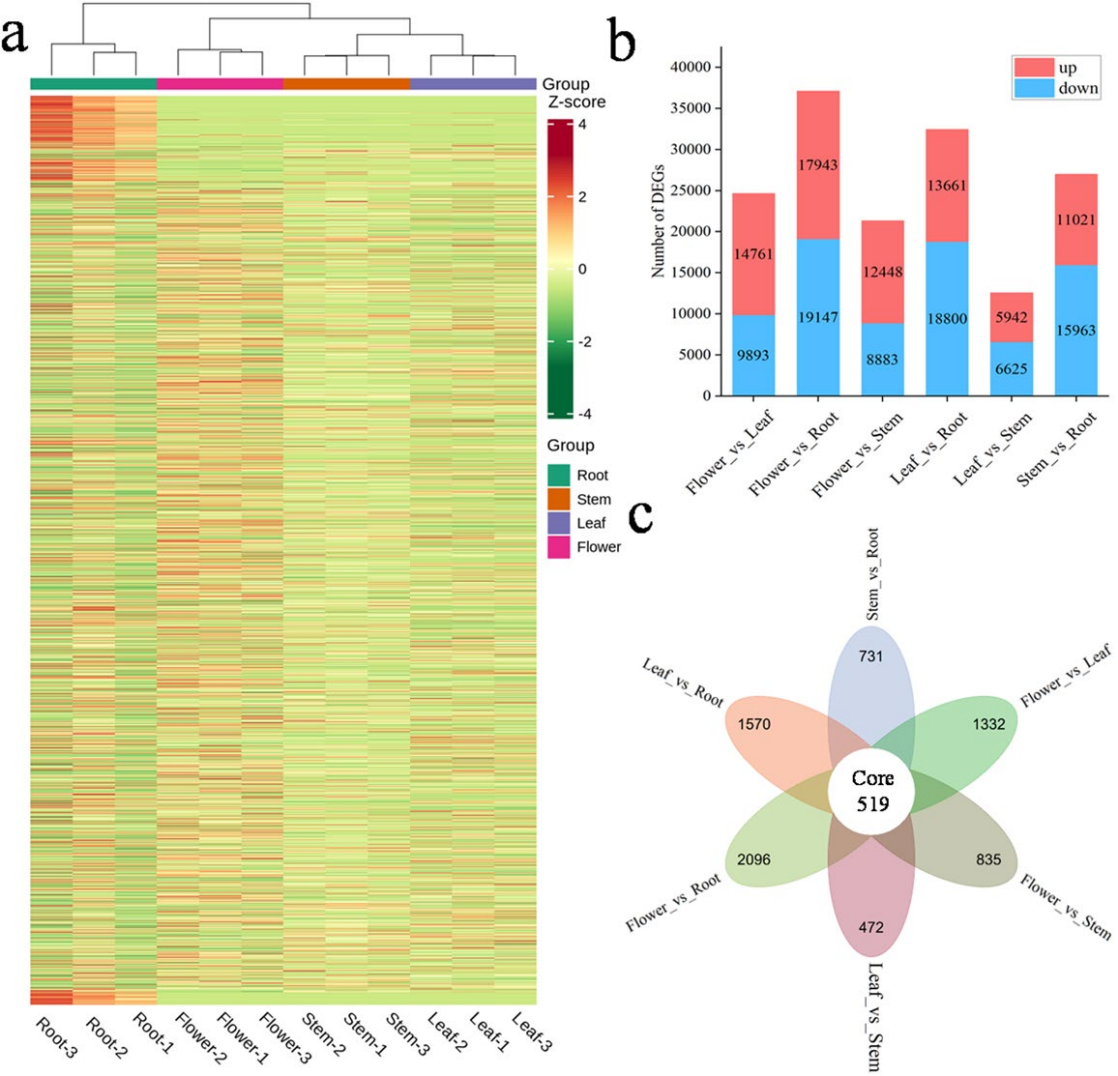
Identification of DEGs. (**a**) Hierarchical clustering heatmap of DEGs. The FPKM values are color-coded and normalized by the Z score. (**b**) Number of DEGs in the six pairs of comparisons. (**c**) Venn diagram of DEGs in the six pairs of comparisons.

A total of 53610 DEGs were identified across the six pairs of comparisons on the basis of the criteria of |log2 fold change| ≥ 1 and FDR < 0.05. The hierarchical clustering analysis of DEGs is shown in Fig. 5a, with detailed information provided in Additional Table 5. The analysis revealed significant differences in the expression patterns of DEGs across the four tissues. Figure 5b shows the number of DEGs identified through pairwise comparisons between different tissues. Specifically, the comparison of flowers to leaves revealed 24654 DEGs (14761 upregulated and 9893 downregulated). A comparison of flowers and roots revealed 37090 DEGs (17943 upregulated and 19147 downregulated). The comparison gulated and 6625 downregulated). Finally, the comparison of stems to roots revealed 26984 DEGs (11021 upregulated and 15963 downregulated). Figure 5c shows that 519 DEGs were common among all six pairs of comparisons. KEGG enrichment of the 519 common DEGs revealed a pronounced metabolic emphasis, with 55.45% of the genes mapping to general metabolic pathway and 35.15% to secondary-metabolite biosynthesis. Within the latter, flavonoid biosynthesis (*CHS*, *F3H*, *ANS*, *CHI*, *DFR*) and phenylpropanoid biosynthesis (*PAL*, *C4H*, *4CL* and others) were explicitly represented, confirming transcriptional activation of the flavonoid backbone across all tissues. Concurrently, defence-related categories-plant-pathogen interaction (13.37%), MAPK signalling (6.93%) and hormone signal transduction (10.4%)—were significantly enriched, underscoring a coordinated reprogramming of secondary metabolism and stress signalling within this core gene set (See Additional Fig. 4).

**Fig. 6:**
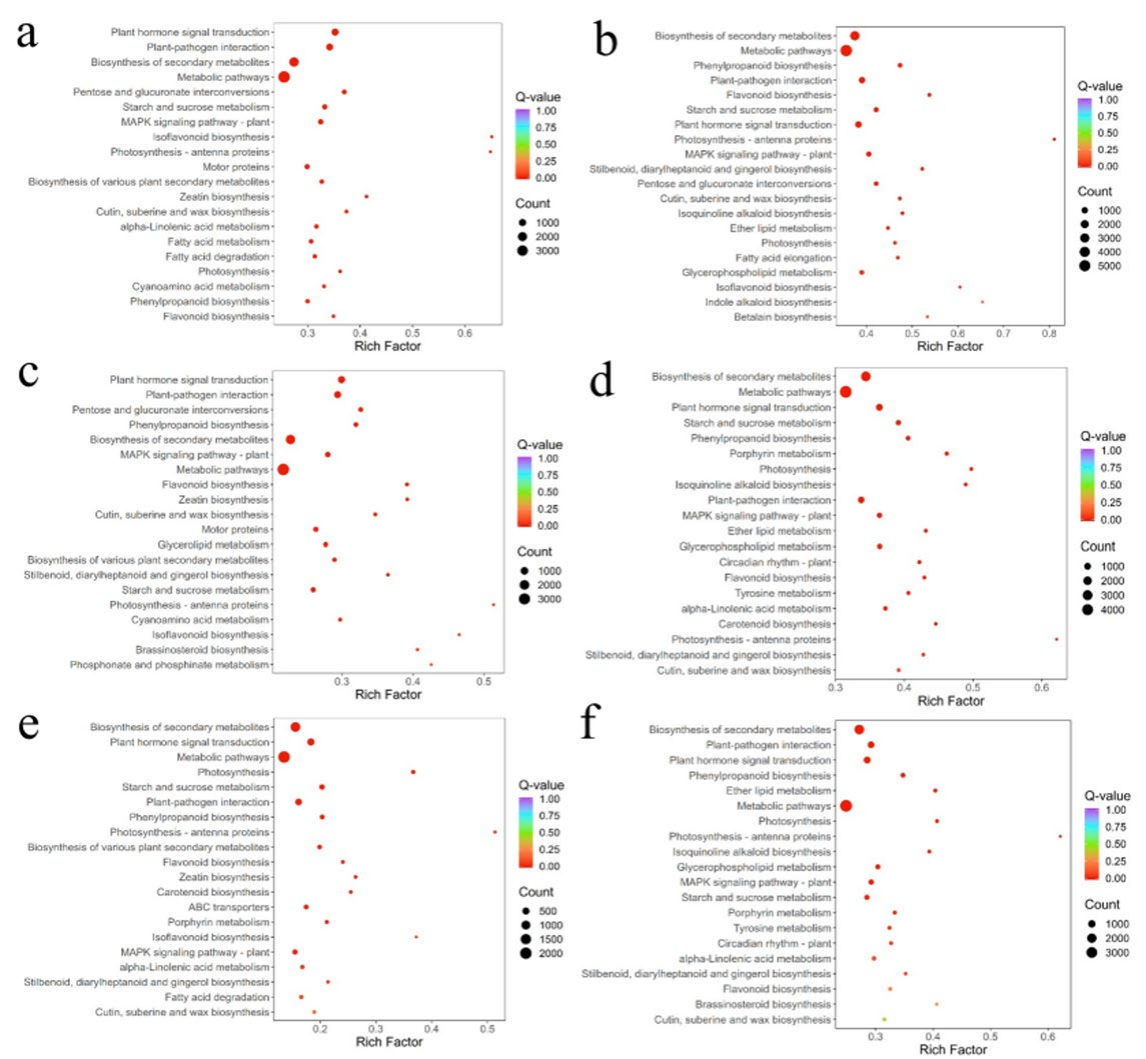
KEGG enrichment analysis of DEGs from the six pairs of comparisons. The horizontal axis represents the Rich Factor corresponding to each pathway. The vertical axis represents the pathway name. The color of the dots indicates the Q-value. The size of the dots corresponds to the number of DEGs. The 20 most significantly enriched pathways are shown in each figure. (**a**) Flower vs. leaf (**b**) flower vs. root (**c**) flower vs. stem (**d**) leaf vs. root (**e**) leaf vs. stem (**f**) stem vs. root. (Enrichment results were obtained from the Kyoto Encyclopedia of Genes and Genomes database and is reproduced with permission, www.kegg.jp/kegg/kegg1.html)

To identify genes associated with flavonoid biosynthesis, the DEGs were subjected to GO functional enrichment analysis (See Additional Fig. 5). In each pairs comparison, the DEGs were categorized into three main categories: biological process (BP), cellular component (CC), and molecular function (MF). In terms of BP, a substantial number of DEGs were enriched in cellular processes, metabolic processes, response to stimulus, and biological regulation. For the CC category, DEGs were predominantly enriched in cellular anatomical entities and protein-containing complexes. With respect to MF, the majority of DEGs were enriched in binding, catalytic activity, transcription regulator activity, and transporter activity. The results of the GO enrichment analysis indicated that the DEGs are significantly enriched in biosynthesis and catabolism, suggesting their potential involvement in flavonoid biosynthesis. Additionally, the DEGs were subjected to KEGG pathway enrichment analysis (Fig. 6). The DEGs were mapped to 96, 96, 100, 81, 97, and 96 KEGG pathways in the comparisons of flower vs. root, flower vs. stem, flower vs. leaf, stem vs. root, leaf vs. root, and leaf vs. stem, respectively. KEGG enrichment across six pairwise comparisons identified the core enzymatic repertoires for each flavonoid-related pathway: phenylpropanoid biosynthesis (ko00940) was represented by *PAL*, *4CL*, *HCT*, *C3′H*, *COMT* and *F5H*; flavonoid biosynthesis (ko00941) by *CHS*, *CHI*, *F3H*, *DFR*, *ANS*, *FLS* and *ANR*; and isoflavonoid biosynthesis (ko00943) by *FNS*, *HIDH*, *VR* and *I2′H*.

### KEGG coenrichment analysis of DEGs and dams

**Fig. 7:**
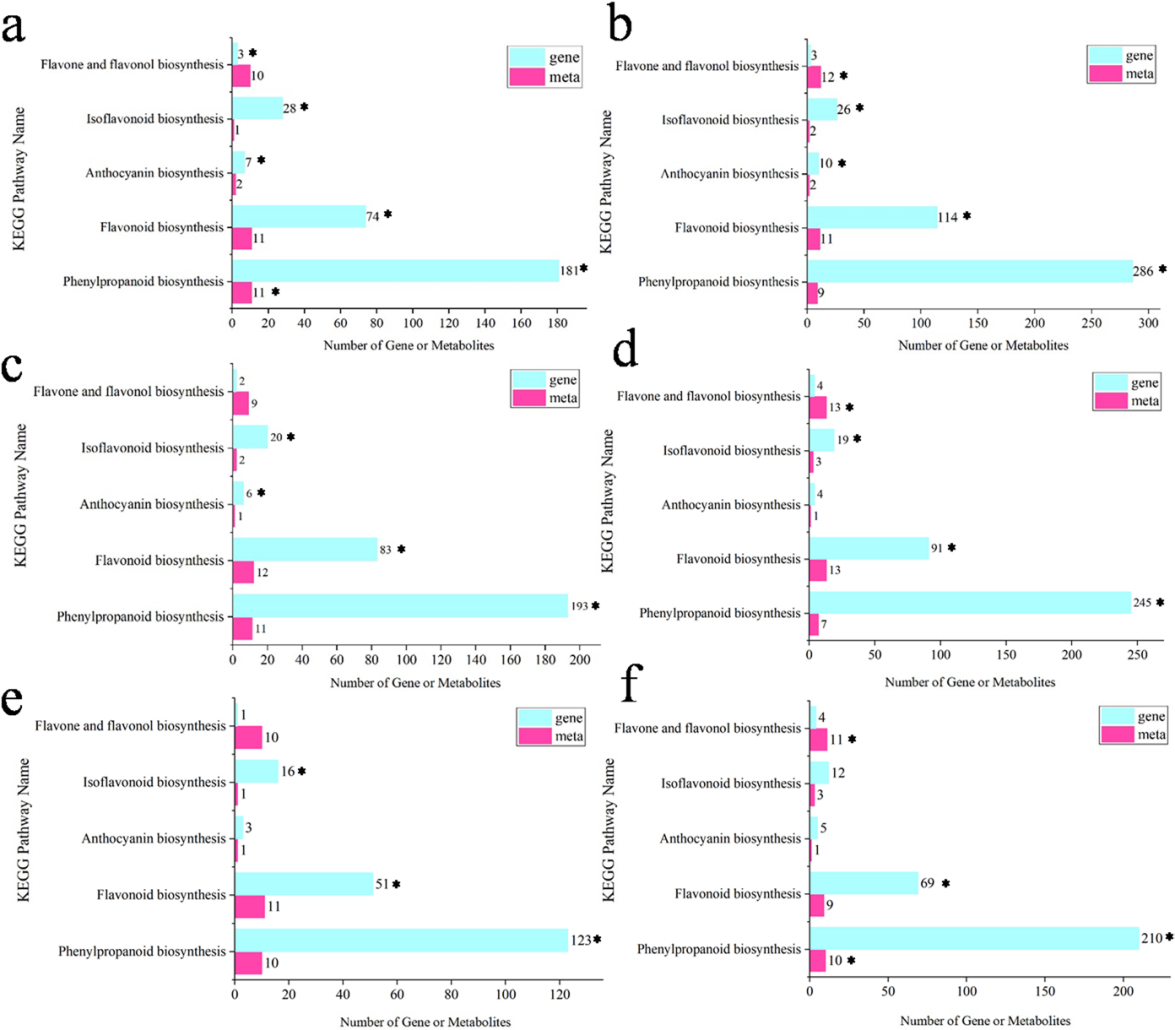
KEGG coenrichment analysis of DEGs and DAMs; “*” represents *P*-values or Q-values < 0.05. (**a**) flower vs. leaf (**b**) flower vs. root (**c**) flower vs. stem (**d**) leaf vs. root (**e**) leaf vs. stem (**f**) stem vs. root.

The coenrichment of DEGs and DAMs in KEGG biosynthetic pathways across six comparison pairs is depicted in Fig. 7. The x-axis represents the number of genes or metabolites, while the y-axis represents the KEGG pathways related to flavonoid biosynthesis (genes are indicated in light blue, and metabolites are indicated in pink). The numbers following the bars in the figure indicate the quantity of enriched genes or metabolites, and the asterisks (*) indicate that the significance threshold for the KEGG pathway is less than 0.05. The pathway with the highest coenrichment of both genes and metabolites is phenylpropanoid biosynthesis (ko00940), followed by flavonoid biosynthesis (ko00941), isoflavonoid biosynthesis (ko00943), flavone and flavonol biosynthesis (ko00944), and lastly anthocyanin biosynthesis (ko00942). The substantial enrichment of the phenylpropanoid biosynthesis pathway (ko00940) across all comparisons highlights its essential role in the flavonoid biosynthetic network. Its significant prominence can be largely attributed to its initial position within the biosynthetic cascade of flavonoids, which establishes it as a key regulatory node. Among these pathways, the significance of enrichment varied between the transcriptome and metabolome across the six pairs of comparisons. For example, in the flower vs. leaf group, all pathways were significantly enriched in the transcriptome, but only the phenylpropanoid biosynthesis pathway was significantly enriched in the metabolome. It should be noted that in this study, we conducted KEGG pathway enrichment analysis on DEGs and DAMs, focusing on pathway related to flavonoid biosynthesis. Although we did not apply stringent statistical thresholds, these pathways are of significant biological importance. Despite not meeting the stringent statistical criteria for enrichment, these pathways may still have significant biological relevance. To substantiate their roles within the studied biological systems, future research endeavors should prioritize the validation of these marginally significant pathways through rigorous experimental designs. The coenrichment of DEMs and DEGs highlights the close regulatory interplay between the transcriptome and metabolome. These coenriched genes and metabolites thus represent prime candidates for further experimental validation.

### Construction of a correlation network diagram between DEGs and dams

**Fig. 8:**
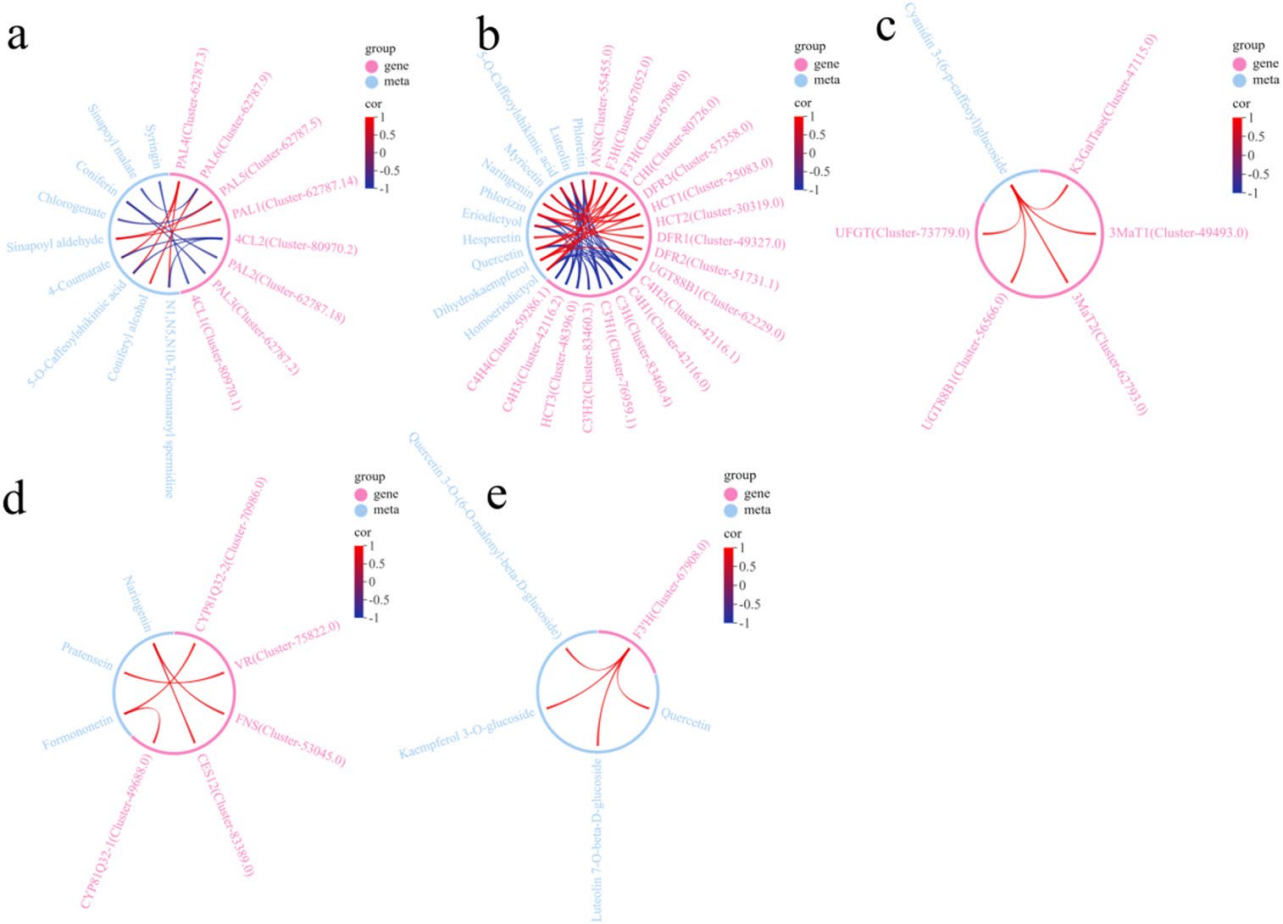
Correlation network diagram of DEGs and DAMs coenriched in KEGG pathways. (**a**) ko00940; (**b**) ko00941; (**c**) ko00942; (**d**) ko00943; (**e**) ko00944. *PAL*: Phenylalanine Ammonia Lyase; *4CL*: 4-Coumarate: CoA Ligase; *HCT*: Hydroxycinnamoyl-CoA: Shikimate/Quinate Hydroxycinnamoyl Transferase; *C4H*: Cinnamate 4-Hydroxylase; *DFR*: Dihydroflavonol 4-Reductase; *ANS*: Anthocyanidin Synthase; *UGT88B1*: UDP-Glucosyltransferase 88B1; *F3H*: Flavanone 3-Hydroxylase; *F3’H*: Flavonoid 3’-Hydroxylase; *C3’H*: p-Coumaroyl ester 3’-hydroxylase; *CHI*: Chalcone Isomerase; *C3H*: p-Coumarate 3-hydroxylase; *K3GalTase*: Kaempferol 3-O-beta-D-galactosyltransferase; *3MaT*: 3-O-Malonyltransferase; *UFGT*: Flavonoid 3-O-glucosyltransferase; *CYP81Q32*: Cytochrome P450 81Q32; *FNS*: Flavanone Synthase; *VR*: Vestitone Reductase; *CES12*: Carboxylesterase 12.

Pearson correlation coefficients were calculated for DAMs and DEGs that were co-enriched in each KEGG pathway, with a transcriptome Q-value less than 0.05. Specifically, DAMs and DEGs from all six comparison pairs were combined for each KEGG pathway to perform the correlation analysis^28^. A correlation network diagram was constructed and is shown in Fig. 8. The analysis revealed significant correlations between a total of 36 DEGs and 25 DAMs (|R|>0.8, *P* < 0.05). In the phenylpropanoid biosynthesis pathway (ko00940) (Fig. 8a), a total of 8 DEGs were significantly correlated with 9 DAMs. Specifically, the metabolites 4-coumarate, sinapoyl aldehyde, 5-O-caffeoylshikimic acid, coniferyl alcohol, and N1,N5,N10-tricoumaroyl spermidine exhibited significant positive correlations with the genes *PAL1* (Cluster-62787.14), *PAL4* (Cluster-62787.3), *PAL5* (Cluster-62787.5), and *PAL6* (Cluster-62787.9). All other genes, except for *PAL4* (Cluster-62787.3), were significantly negatively correlated with these metabolites. In the flavonoid biosynthesis pathway (ko00941) (Fig. 8b), a significant correlation was observed between 18 DEGs and 11 DAMs. The genes *HCT1* (Cluster-25083.0), *HCT2* (Cluster-30319.0), *DFR1* (Cluster-49327.0), *DFR2* (Cluster-51731.1), *DFR3* (Cluster-57358.0), *ANS* (Cluster-55455.0), *UGT88B1* (Cluster-62229.0), *F3H* (Cluster-67052.0), *F3’H* (Cluster-67908.0), and *CHI* (Cluster-80726.0) presented significant positive correlations with the aforementioned 11 metabolites. The nine genes, such as *C4H1* (Cluster-42116.0), exhibited significant negative correlations with these metabolites. In the anthocyanin biosynthesis pathway (ko00942) (Fig. 8c), only one DAM was significantly positively correlated with five DEGs, including cyanidin 3-(6-p-cafeoyl) glucoside, *K3GAT* (Cluster-47115.0), *3MaT1* (Cluster-49493.0), *UGT88B1* (Cluster-56566.0), *3MaT2* (Cluster-62793.0), and *UFGT* (Cluster-73779.0). In the isoflavonoid biosynthesis pathway (ko00943) (Fig. 8d), several metabolites presented significant positive correlations with specific genes. Specifically, naringenin was significantly positively correlated with *CES12* (Cluster-83389.0) and *FNS* (Cluster-53045.0); pratensein with *VR* (Cluster-75822.0); and formononetin with *CYP81Q32-2* (Cluster-70986.0) and *CYP81Q32-1* (Cluster-49688.0). In the flavone and flavonol biosynthesis pathway (ko00944) (Fig. 8e), the metabolites quercetin 3-o-(6-o-malonyl-beta-D-glucoside), kaempferol 3-o-glucoside, luteolin 7-o-beta-D-glucoside, and quercetin were significantly positively correlated with *F3’H* (Cluster-67908.0). On the basis of the above analysis, we propose that these differentially expressed genes likely play crucial roles in regulating flavonoid biosynthesis in *Eupatorium lindleyanum*.

### Construction of the flavonoid biosynthesis regulatory network in *Eupatorium lindleyanum*

**Fig. 9:**
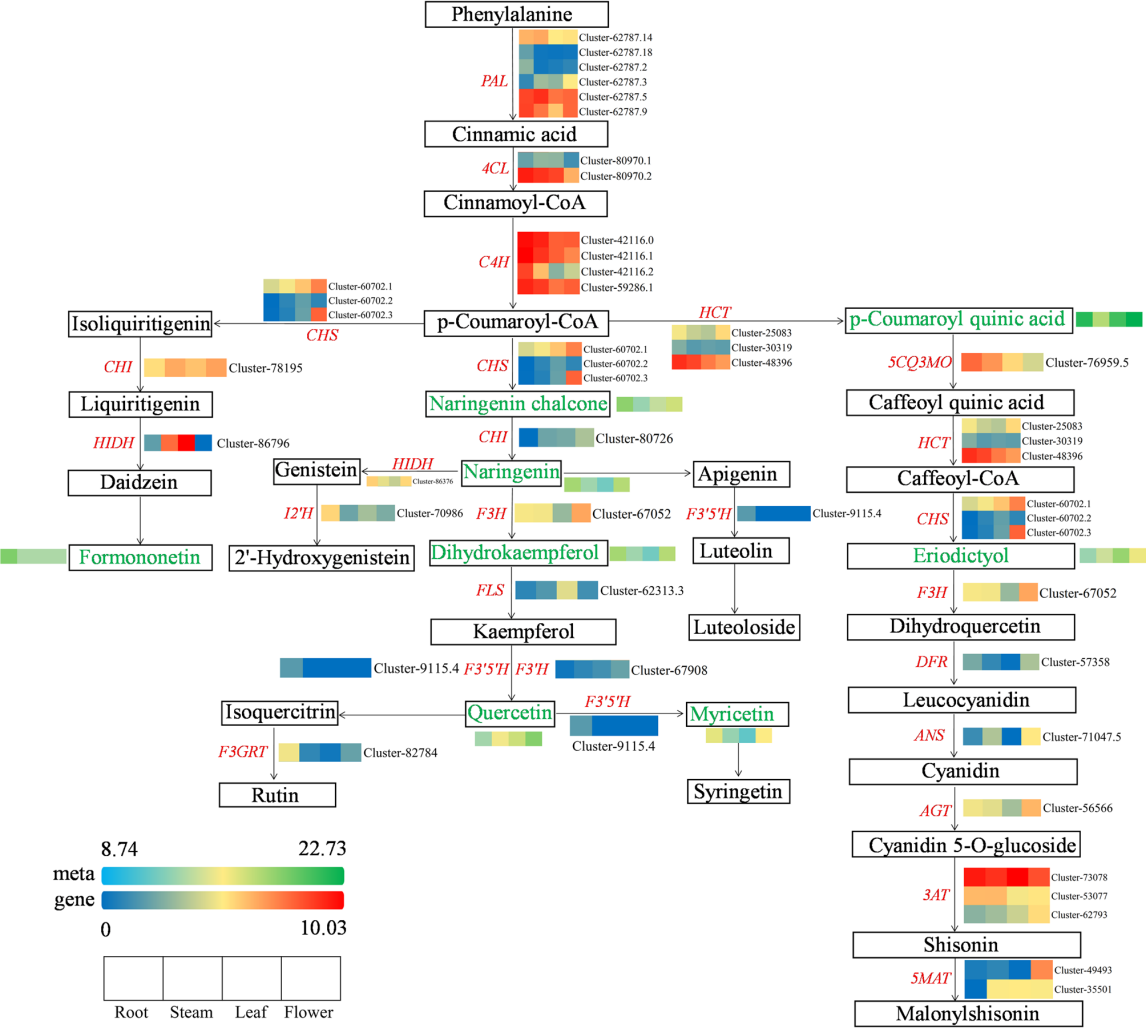
Predicted flavonoid biosynthesis pathway in different tissues of *Eupatorium lindleyanum.* The green words in the box represent DAMs, and the red words between metabolites represent DEGs. *PAL*: Phenylalanine ammonia-lyase; *4CL*: 4-coumarate-CoA ligase; *C4H*: Trans-cinnamate 4-monooxygenase; *CHS*: Chalcone Synthase; *CHI*: Chalcone Isomerase; *HIDH*: 2-hydroxyisoflavanone dehydratase; *I2’H*: Isoflavone/4’-methoxyisoflavone 2’-hydroxylase; *F3H*: Flavanone 3-Hydroxylase; *FLS*: Flavonol Synthase; *F3’5’H*: Flavonoid 3’,5’-hydroxylase; *F3’H*: Flavonoid 3’-monooxygenase; *F3GRT*: Flavonol-3-O-glucoside L-rhamnosyltransferase; *HCT*: Shikimate O-Hydroxycinnamoyltransferase; *DFR*: Dihydroflavonol 4-Reductase; *ANS*: Anthocyanidin synthase; *AGT*: Anthocyanidin 5,3-O-Glucosyltransferase; *FOMT*: Flavonoid O-Methyltransferase; *5CQ3MO*: 5-O-(4-coumaroyl)-D-quinate 3’-monooxygenase; *3AT*: 3-O-glucoside 6’’-O-acyltransferase; *5MAT*: 5-O-glucoside-6’’’-O-malonyltransferase.

To elucidate the molecular mechanisms underlying flavonoid biosynthesis in different tissues of *Eupatorium lindleyanum*, we constructed a heatmap of the flavonoid biosynthesis pathway on the basis of the KEGG coenrichment and correlation analysis results of the DEGs and DAMs (Fig. 9). These results indicated that six *PAL* genes, two *4CL* genes, and four *C4H* genes play regulatory roles in the initial stage of flavonoid biosynthesis, specifically from phenylalanine to p-coumaroyl-CoA. During the biosynthesis of isoflavones, p-coumaroyl-CoA is sequentially converted into isoliquiritigenin, then to liqiritigenin, and finally to daidzein through the catalytic actions of specific enzymes, including *CHS*, *CHI*, and *HIDH*. In *Eupatorium lindleyanum*, naringenin is converted into 2’-hydroxygenistein via the catalytic actions of *HIDH* and *I2’H* in another pathway of isoflavone biosynthesis. In the biosynthetic pathway of flavonoids and flavonols, p-coumaroyl-CoA is sequentially metabolized into naringenin chalcone, naringenin, dihydrokaempferol, kaempferol, and quercetin, culminating in the formation of rutin and syringenin. The key genes regulating this pathway include *CHS*, *CHI*, *F3H*, *FLS*, *F3’5’H*,* F3’H*, and *F3GRT*. The key metabolites in the anthocyanin biosynthesis pathway include caffeoyl-CoA, eriodictyol, dihydrokaempferol, leucocyanidin, cyanidin, cyanidin 5-O-glucoside, shisonin, and malonylshisonin. These compounds were synthesized through the catalytic actions of *CHS*, *F3H*, *DFR*, *ANS*, *AGT*, *3AT*, and *5MAT*, respectively.

Additionally, our analysis revealed that four differentially accumulated metabolites, namely, naringenin chalcone, naringenin, dihydrokaempferol, and formononetin, significantly accumulated in the roots of *Eupatorium lindleyanum*. Furthermore, the metabolite eriodictyol significantly accumulated in the leaves, whereas quercetin significantly accumulated in the flowers. Conversely, the accumulation level of the metabolite p-coumaroyl quinic acid significantly decreased in the stem. Notably, the genes *PAL*, *C4H*, *F3’5’H*, *F3GRT*, *CHI*, *HCT*, and *5CQ3MO* presented significantly higher expression levels in the roots than in the other tissues. In contrast, the *CHS*,* F3H* and *5MAT* genes were highly expressed in flowers. Additionally, the expression levels of the *FLS*, *F3′H*,* DFR*, *ANS*, and *AGT* genes were significantly elevated in the leaves, this also explains why leaves are rich in flavonols and flavones as mentioned earlier. These results underscore the intricate interplay between gene expression and metabolite accumulation across different tissues of *Eupatorium lindleyanum*.

### Analysis of transcription factors associated with flavonoid biosynthesis

**Fig. 10:**
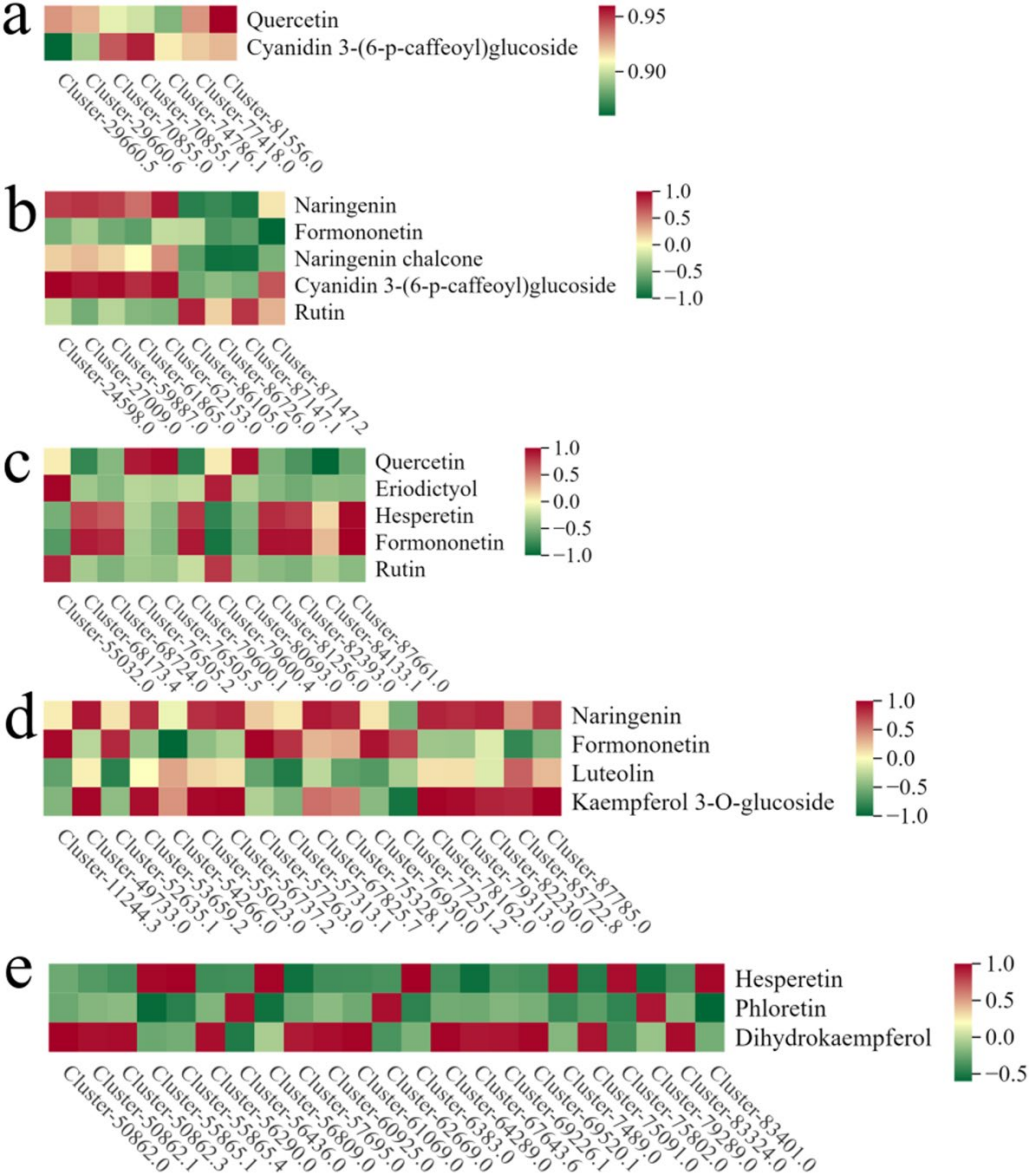
Correlation analysis between transcription factors and DFMs in *Eupatorium lindleyanum* (|R|>0.8, *P* < 0.05); (**a**) *AP2/ERF*; (**b**) *NAC*; (**c**)*WRKY*; (**d**) *MYB*; (**e**) *bHLH.*

The abundance of flavonoid metabolites that differ among the four tissues of *Eupatorium lindleyanum* is regulated by transcription factors^30^. In this study, we identified differentially expressed transcription factor families (|log2fold change| ≥ 1 and FDR < 0.05) and their correlations with different flavonoid metabolites (|R| > 0.8, *P* < 0.05). *AP2/ERF* transcription factors: A total of 7 *AP2/ERF* transcription factors were identified. All five members of this family were significantly positively correlated with quercetin and cyanidin 3-(6-p-caffeoyl)glucoside. *NAC* transcription factors: Nine *NAC* transcription factors were identified. Cluster-24598.0 and the other six genes presented significant positive correlations with naringenin, cyanidin 3-(6-p-caffeoyl)glucoside, and rutin. Conversely, Cluster-86105.0 and three others showed significant negative correlations with naringenin, formononetin, and naringenin chalcone. *WRKY* transcription factors: Twelve *WRKY* transcription factors were identified. Cluster-55032.0 and 10 others exhibited significant positive correlations with quercetin, eriodictyol, hesperetin, formononetin, and rutin. In contrast, Cluster-68173.4 and three others were significantly negatively correlated with quercetin, hesperetin, and formononetin. *MYB* transcription factors: Eighteen *MYB* transcription factors were identified. Cluster-11244.3 and 14 others exhibited significant positive correlations with naringenin, formononetin, kaempferol 3-O-glucoside, and rutin. Conversely, Cluster-52635.1 and four other genes presented significant negative correlations with formononetin, luteolin, kaempferol 3-O-glucoside, and rutin. *bHLH* transcription factors: Twenty-three *bHLH* transcription factors were identified. Cluster-50862.0 and 22 exhibited significant positive correlations with hesperetin, phloretin, and dihydrokaempferol. In contrast, Cluster-55865.1 and six others exhibited significant negative correlations with these metabolites (Fig. 10). The differences in the expression patterns of the transcription factors among the four tissues are presented in Additional Fig. 6. These results suggest that these transcription factors play important roles in regulating flavonoid biosynthesis in different tissues of *Eupatorium lindleyanum*.

### qRT‒PCR validation of candidate genes in the flavonoid biosynthesis pathway

**Fig. 11:**
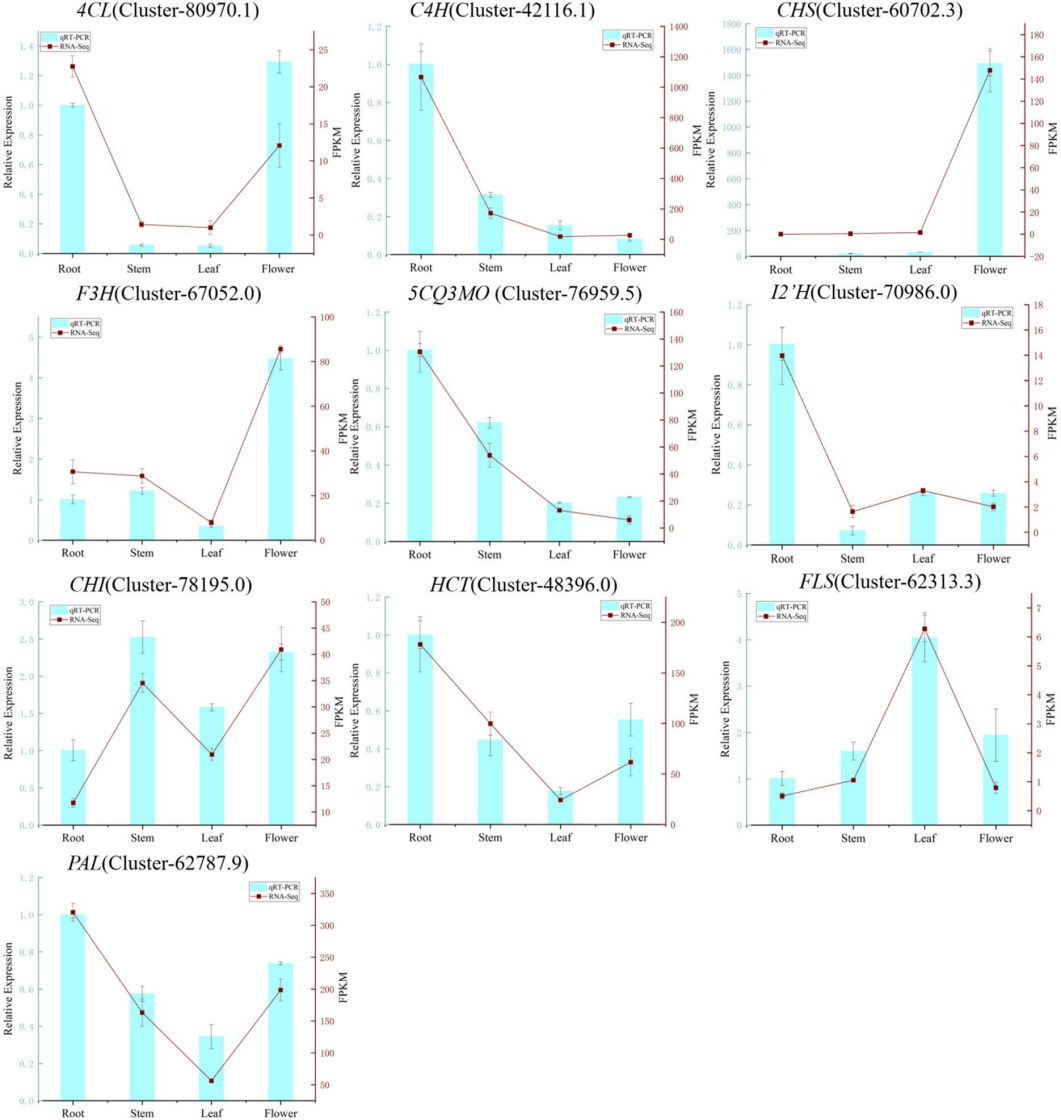
qRT‒PCR validation of 10 candidate genes. The horizontal axis represents the four tissues of *Eupatorium lindleyanum*, the left vertical axis represents the relative expression levels of genes determined via qRT‒PCR, and the right vertical axis represents the FPKM values of the genes.

To verify the accuracy of the transcriptome data, 10 candidate genes were selected for qRT‒PCR validation. As shown in Fig. 11, the relative expression levels of these genes were consistent across the four tissues of *Eupatorium lindleyanum* compared with the FPKM values derived from RNA-Seq. This result indicates that the transcriptome sequencing data are accurate.

## Discussion

Flavonoids are a diverse group of secondary metabolites widely present in plants that exhibit significant physiological activities and substantial application value^31^. *Eupatorium lindleyanum*, a medicinal plant, is rich in flavonoids and has been demonstrated to exhibit anti-inflammatory, antibacterial, antitumour, and antioxidant activities^32^. However, the biosynthetic mechanisms and functional gene screening of flavonoids in this plant have not yet been fully elucidated, which limits the comprehensive understanding and utilization of these valuable compounds. In this study, an integrative approach combining metabolomics, transcriptomics, and bioinformatics was employed to elucidate the flavonoid biosynthesis pathway and related genes in *Eupatorium lindleyanum*^33^. The integration of metabolomic and transcriptomic analyses has provided profound insights into the tissue-specific biosynthesis of flavonoids. However, in the co-enrichment analysis of transcriptome and metabolome, we also observed that the significance levels of certain KEGG pathways for metabolomic data were greater than 0.05, which may not directly reflect the overall activity status of the metabolic pathways. Firstly, due to the lag in metabolic regulation, transcriptional changes often precede metabolic changes, and the accumulation of metabolites may require more time to reach the detection threshold. Consequently, even if the expression of key enzyme genes has changed, the concentration of metabolites may not have undergone significant alterations^34^. Secondly, the stability of metabolites and detection limitations may also affect the significance of the results. Some metabolites may be unstable during sample collection and processing, prone to degradation or transformation, or not accurately quantified due to the sensitivity constraints of detection technologies^35^. Additionally, the comparative analysis of different plant tissues may not have targeted the pathway in question, or the pathway may not be active in specific tissues, which could also lead to a significance level greater than 0.05 for metabolomic data^36^. Nonetheless, even if the changes in some metabolites are not statistically significant, they may still possess important biological significance. Therefore, further experimental validation and biological interpretation are required to ascertain the biological relevance of these changes. These results reveal the complex regulatory mechanisms underlying the tissue-specific production of these secondary metabolites, providing valuable insights for targeted metabolic engineering and breeding strategies.

In this study, we identified 330 differentially accumulated flavonoid metabolites in *Eupatorium lindleyanum*, including anthocyanidins, aurones, chalcones, flavanols, flavanones, flavanonols, flavones, flavonols, isoflavones, and other flavonoids. This finding is consistent with those of previous studies^3^. Principal component analysis (PCA) and hierarchical clustering analysis revealed distinct tissue-specific signatures of these metabolites in roots, stems, leaves, and flowers. All four tissues contained the same classes of flavonoids, but the relative abundance of these metabolites varied significantly among the tissues. In roots and stems, the relative abundance of differentially accumulated flavonoids was generally low, with only a few metabolites showing high abundance. For example, cicerin-7-O-glucoside-6’’-malonate, formononetin, and diosmetin-6-C-glucoside were highly abundant in roots. Similarly, 3’-methyl-2’,4’,6’-trihydroxydihydrochalcone, 3,3’,5-trihydroxy-4’,7-dimethoxyflavanone, and isorhamnetin 3-(2’’-acetylglucoside) were elevated in stems. In contrast, the leaves and flowers contained relatively high levels of most flavonoids, with particularly elevated concentrations observed in the leaves. This pattern suggests that the accumulation of flavonoids is tissue-specific and may be regulated by specific genes^37^. The distinct metabolic profiles observed across different tissues reflect their specialized physiological roles and adaptive responses to environmental cues. Roots are involved primarily in nutrient absorption and storage, whereas leaves and flowers are exposed to relatively high levels of environmental stressors, such as ultraviolet (UV) radiation and herbivory. The elevated levels of flavonoids in leaves and flowers likely play dual roles: as a protective mechanism against oxidative stress and UV-induced damage and as floral attractants to facilitate pollination processes^38–41^. Therefore, we propose that the distribution pattern of the flavonoid content in *Eupatorium lindleyanum* is under the regulatory control of specific genes. Future research should focus on identifying these regulatory genes and elucidating their mechanisms of action.

Flavonoids, as secondary metabolites in plants, are predominantly regulated by structural genes in the biosynthetic pathway^42^. In this study, the integration of transcriptomic and metabolomic analyses revealed that a total of 27 structural genes encoding 52 unigenes were involved in the biosynthesis of flavonoids in *Eupatorium lindleyanu*m (See Additional Table 6). The genes were classified into four groups on the basis of their involvement in distinct biosynthetic pathway branches. Specifically, Group I genes are involved in the biosynthesis of phenylpropanoids, including *PAL*, *4CL*, *C3H*, *C3’H*, and *C4H*. The Group II genes are associated with the biosynthesis of flavones and flavonols and include *CHS*, *CHI*, *F3H*, *FLS*, *F3’5’H*, *F3’H*, *F3GRT*, *HCT*, *5CQ3MO*, *FNS*, and *UGT88B1*. Group III genes participate in the biosynthesis of isoflavones, including *HIDH*, *I2’H*, and *VR*. Finally, Group IV genes are involved in the biosynthesis of anthocyanins, including *ANS*, *DFR*, *AGT*, *3AT*, *5MAT*, *K3GalTase*, *3MaT*, and *UFGT*. In previous studies, these genes have been characterized thoroughly and are well documented to play crucial roles in the biosynthesis of plant flavonoids. For example, in different tissues of *Polygonatum cyrtonema* Hua, genes such as *HCT*, *4CL*, *PAL*, *C3’H*, *CHI*, *FLS*, *F3’H*, *CHS*, *FNS*, and *DFR* were identified as core regulatory genes involved in flavonoid biosynthesis via multiomics jiont technology^43^. Similar results have also been reported for *Punica granatum*^44^, *Fragaria vesca*^45^, *Malus domestica*^46^, *Vitis labrusca*^47^, *Asparagus officinalis*^48^, *Scutellaria baicalensis*^17^, *Carthamus tinctorius*^49^, and other horticultural or medicinal plants. Remarkably, the core structural genes display a high degree of functional conservation across medicinal Asteraceae. In *Coreopsis tinctoria*, integrated transcriptome–metabolome analyses of two floral colour morphs revealed that elevated expression of *F3H* and *3GT* is directly associated with anthocyanin accumulation^50^. Likewise, developmental-stage-specific regulation of *HCT*, *C4H*, and *F3′H* in *Chrysanthemum morifolium* tightly correlates with the temporal biosynthesis of kaempferol and its acylated derivatives^51^. While *Eupatorium lindleyanum* shares the conserved phenylpropanoid–flavonoid “toolkit” with these congeners, *Coreopsis tinctoria* enhances *F3′5′H* activity to channel flux toward cyanidin-type anthocyanins, whereas *Chrysanthemum morifolium CYP81E*-mediated hydroxylation and methylation to produce kaempferol-acylated products. These findings underscore that Asteraceae species deploy lineage-specific terminal decorations on a conserved flavonoid scaffold, providing a mechanistic basis for metabolic diversification within the family. Additionally, the functions of these genes in the flavonoid biosynthesis pathway have been widely preliminarily confirmed in other plants^52,53^. *PAL*, *C4H*, and *4CL* are key enzymes in the phenylpropanoid pathway that catalyze the conversion of phenylalanine to 4-coumaroyl-CoA, which provides the precursor for flavonoid biosynthesis. *C3H* and *C3’H* participate in the shikimate pathway, further modifying the structure of flavonoids. *CHS* and *CHI* are core enzymes in the flavonoid biosynthesis pathway, with *CHS* catalyzing the formation of chalcone from 4-coumaroyl-CoA and malonyl-CoA, and *CHI* converting chalcone to flavanone. *F3H* further catalyzes the conversion of flavanone to dihydroflavonols, providing a key intermediate for flavonoid synthesis. *F3’H* and *F3’5’H* increase the structural diversity of flavonoids through hydroxylation reactions. *FLS* catalyzes the formation of flavonols from dihydroflavonols, whereas *DFR* converts dihydroflavonols to colorless anthocyanidins. *ANS* and *ANR* catalyze the synthesis and reduction of anthocyanidins, respectively, and participate in the biosynthesis of anthocyanins and flavanols. *UGT88B1* and *UFGT* increased the water solubility and stability of flavonoids through glycosylation reactions. Additionally, *3AT*, *5MAT*, and *K3GalTase* further modulate the biological activity of flavonoids through acetylation, methylation, and glucosylation^54–57^. The coordinated action of these genes within the flavonoid biosynthesis pathway dictates the tissue-specific distribution of flavonoids in *Eupatorium lindleyanum*.

The involvement of transcription factors (TFs) in the regulation of flavonoid biosynthesis has been well reported in various plant species. In this study, we identified several TF families, including *AP2/ERF*, *NAC*, *WRKY*, *MYB*, and *bHLH*, that were significantly correlated with the differential accumulation of flavonoids. These TFs likely play crucial roles in modulating the expression of structural genes involved in flavonoid biosynthesis. For example, *MYB* and *bHLH* TFs are known to regulate the expression of *CHS*, *CHI*, and *DFR*, which are critical for the synthesis of flavonoids and anthocyanins^58,59^. Furthermore, the significant correlation between these TFs and flavonoid metabolites in different tissues suggests their tissue-specific regulatory functions. To exemplify, *AP2/ERF* TFs, which are positively correlated with quercetin and cyanidin 3-(6-p-caffeoyl)glucoside, may be involved in the regulation of flavonoid production^60,61^. The identification of these TFs provides potential targets for genetic engineering to increase flavonoid production in *Eupatorium lindleyanum*.

By integrating metabolomic and transcriptomic datasets, we constructed a provisional “transcription factor–structural gene–metabolite” network that underpins tissue-specific flavonoid biosynthesis in *Eupatorium lindleyanum*. In flowers, *AP2/ERF* and *MYB* TFs up-regulate *CHS* and *F3H*, channeling carbon toward quercetin and anthocyanin glycosides; in leaves, *WRKY* and *bHLH* members drive *FLS* and *F3′H* expression, leading to the accumulation of flavonols and flavones with antioxidant activity; in roots, *NAC* and *bHLH* TFs enhance *PAL* and *HIDH*, specifically enriching isoflavonoids with defensive roles. In contrast, stems exhibit metabolic inertia, correlating with high *NAC* abundance and the absence of anthocyanin-activating *MYBs*. This framework suggests that aerial tissues prioritize ecologically functional flavonols/anthocyanins, whereas subterranean tissues favor stress-related isoflavonoids, providing molecular targets for targeted manipulation of medicinal flavonoid distribution in molecular breeding programs.

The findings of this study have significant implications for understanding flavonoid biosynthesis in *Eupatorium lindleyanum* and provide a foundation for future research and applications. The identification of key regulatory genes and TFs involved in flavonoid biosynthesis offers potential targets for genetic engineering to increase the production of bioactive flavonoids, which have important medicinal and nutraceutical properties^62^. Furthermore, the tissue-specific accumulation of flavonoids and the differential expression of genes suggest that targeted breeding or tissue culture strategies could be employed to optimize the production of specific flavonoids. In conclusion, this study provides a comprehensive analysis of the metabolome and transcriptome profiles of *Eupatorium lindleyanum*, revealing the tissue-specific accumulation of flavonoids and the differential expression of genes involved in their biosynthesis. The identification of key regulatory genes and TFs, along with the reconstruction of the flavonoid biosynthesis pathway, offered valuable insights into the molecular mechanisms underlying flavonoid production. Future research should focus on functional validation of these genes and TFs through genetic and biochemical approaches, as well as exploring their potential applications in plant breeding and biotechnology.

## Conclusion

In this study, different tissues of fresh *Eupatorium lindleyanum* were used as experimental materials to elucidate the molecular mechanism of flavonoid biosynthesis on the basis of the tissue-specific distribution of flavonoid content. A total of 330 differentially accumulated flavonoid metabolites were identified in the roots, stems, leaves, and flowers of *Eupatorium lindleyanum*. Through correlation analysis of the DEGs and metabolites, 36 genes were screened for significant correlations with these flavonoid metabolites. Finally, by integrating the transcriptomic and metabolomic data, 27 candidate structural genes involved in flavonoid biosynthesis were identified. Additionally, through correlation analysis, 69 transcription factors from five major categories were predicted to regulate flavonoid biosynthesis by modulating the expression of these structural genes. Overall, this study provides a comprehensive foundation for elucidating the molecular mechanisms underlying flavonoid biosynthesis in *Eupatorium lindleyanum* and offers candidate genes for the development of new cultivars with increased flavonoid contents.

## Supplementary Information

Below is the link to the electronic supplementary material.

Supplementary Material 1

Supplementary Material 2

Supplementary Material 3

Supplementary Material 4

Supplementary Material 5

Supplementary Material 6

Supplementary Material 7

Supplementary Material 8

Supplementary Material 9

Supplementary Material 10

Supplementary Material 11

Supplementary Material 12

## Data Availability

The transcriptome sequencing data was deposited in NCBI database under accession number: PRJNA1242508. https://www.ncbi.nlm.nih.gov/sra/PRJNA1242508.

## Abbreviations

UPLC-MS/MS: Ultra Performance Liquid Chromatography-Tandem Mass Spectrometry
DAMs: Differentially accumulated metabolites
DFMs: Differentially accumulated flavonoid metabolites
DEGs: Differentially expressed genes
TFs: Transcription factors
PAL: Phenylalanine Ammonia Lyase
4CL: 4-Coumarate: CoA Ligase
HCT: Hydroxycinnamoyl-CoA: Shikimate/Quinate Hydroxycinnamoyl Transferase
C4H: Cinnamate 4-Hydroxylase
DFR: Dihydroflavonol 4-Reductase
ANS: Anthocyanidin Synthase
UGT88B1: UDP-Glucosyltransferase 88B1
F3H: Flavanone 3-Hydroxylase
F3ʹH: Flavonoid 3ʹ-Hydroxylase
C3ʹH: p-Coumaroyl ester 3ʹ-hydroxylase
CHI: Chalcone Isomerase
C3H: p-Coumarate 3-hydroxylase
K3GalTase: Kaempferol 3-O-beta-d-galactosyltransferase
3MaT: 3-O-Malonyltransferase
UFGT: Flavonoid 3-O-glucosyltransferase
CYP81Q32: Cytochrome P450 81Q32
FNS: Flavanone synthase
VR: Vestitone reductase
CES12: Carboxylesterase 12
