# Supplementary material for: Integrative metabolomic and transcriptomic analyses reveal flavonoid biosynthesis pathway in Eupatorium lindleyanum
Source: Sci Rep. 2025 Dec 4;15:43151. doi: 10.1038/s41598-025-27287-0 (PMC12678412; doi:10.1038/s41598-025-27287-0)
Supplement: Supplementary file 1 — Supplementary Material 1 [file 41598_2025_27287_MOESM1_ESM.pdf]

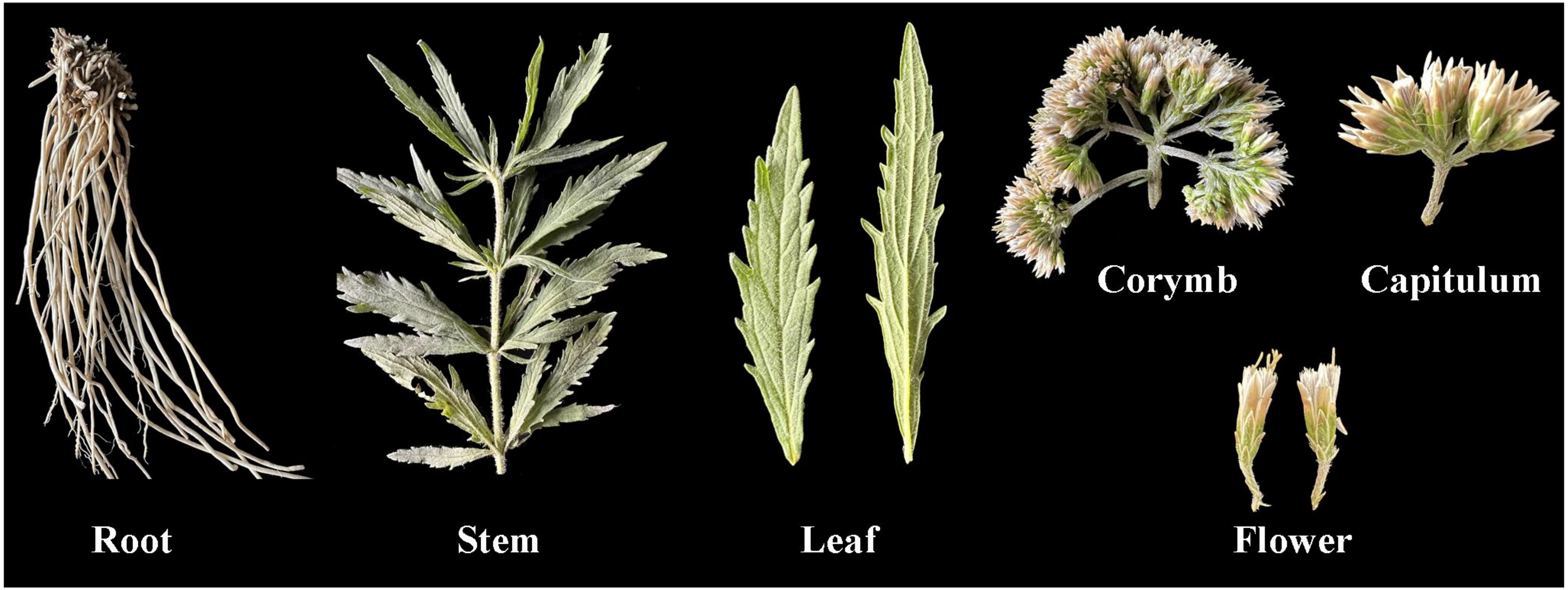

Additional Figure1: Photographs of different tissues of *Eupatorium lindleyanum*, including root, stem, leaf, and flo wer.

The morphological characteristics of *Eupatorium lindleyanum* are consistent with the description in Flora of C hina (http://www.efloras.org/florataxon.aspx?flora_id=2&taxon_id=200023938).

(1) Root and Rhizome: The rhizome is short and bears numerous fibrous roots.

(2) Stem: The stem is erect, branching from the base or simple, and densely covered with long white trichomes or short pubescence.

(3) Leaf: The leaves are elliptic-lanceolate or linear-lanceolate, measuring 3-12 cm in length and 0.5-3 cm in width. The base is cuneate, and the apex is acute. Both surfaces are scabrid, densely covered with long or short, coarse white trichomes, especially along the veins. The leaves are basally 3-veined, with deeply or shallowly serrated margins and are nearly sessile.

(4) Flower: The capitula are numerous and arranged in dense corymbs at the apex of the stem or at the ends of branches. The synflorescence branches and peduncles are purple-red or green, densely covered with short white pubescence. The involucre is campanulate, containing 5 florets. The involucres are arranged in approximately 3 series, imbricate. The outer bracts are short, 1-2 mm in length, lanceolate or broadly lanceolate. The middle and inner bracts are progressively longer, 5-6 mm in length, elliptic or elliptic-lanceolate. All bracts are green or purple-red, with acute apices. The corollas are white, pink, or pale purple-red.
