# Supplementary material for: Integrative metabolomic and transcriptomic analyses reveal flavonoid biosynthesis pathway in Eupatorium lindleyanum
Source: Sci Rep. 2025 Dec 4;15:43151. doi: 10.1038/s41598-025-27287-0 (PMC12678412; doi:10.1038/s41598-025-27287-0)
Supplement: Supplementary file 2 — Supplementary Material 2 [file 41598_2025_27287_MOESM2_ESM.pdf]

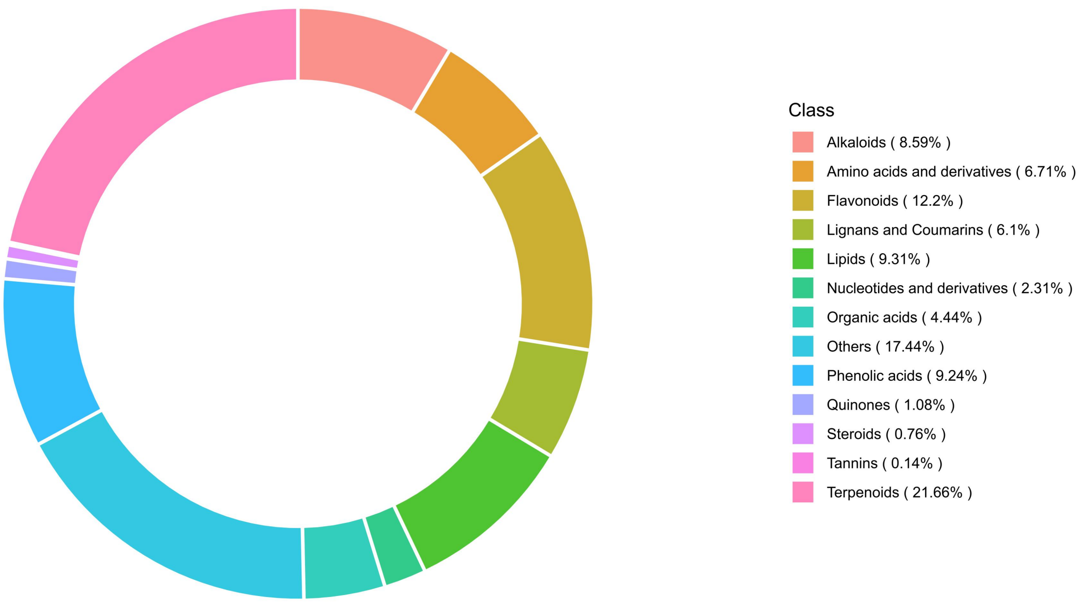

Additional Figure2: Metabolite category composition in four tissues of *Eupatorium lindleyanum*. Percentages indicate the relative abundance of each metabolite category.
