# Supplementary material for: Integrative metabolomic and transcriptomic analyses reveal flavonoid biosynthesis pathway in Eupatorium lindleyanum
Source: Sci Rep. 2025 Dec 4;15:43151. doi: 10.1038/s41598-025-27287-0 (PMC12678412; doi:10.1038/s41598-025-27287-0)
Supplement: Supplementary file 3 — Supplementary Material 3 [file 41598_2025_27287_MOESM3_ESM.pdf]

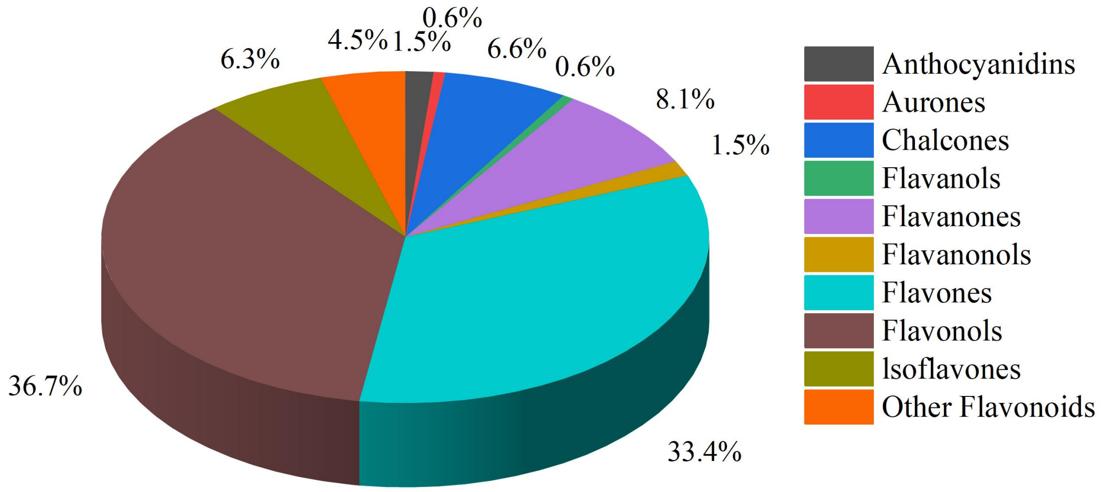

Additional Figure3: Composition and relative abundance of differentially accumulated flavonoid metabolites (DFMs) in *Eupatorium lindleyanum*.
