# Supplementary material for: Integrative metabolomic and transcriptomic analyses reveal flavonoid biosynthesis pathway in Eupatorium lindleyanum
Source: Sci Rep. 2025 Dec 4;15:43151. doi: 10.1038/s41598-025-27287-0 (PMC12678412; doi:10.1038/s41598-025-27287-0)
Supplement: Supplementary file 4 — Supplementary Material 4 [file 41598_2025_27287_MOESM4_ESM.pdf]

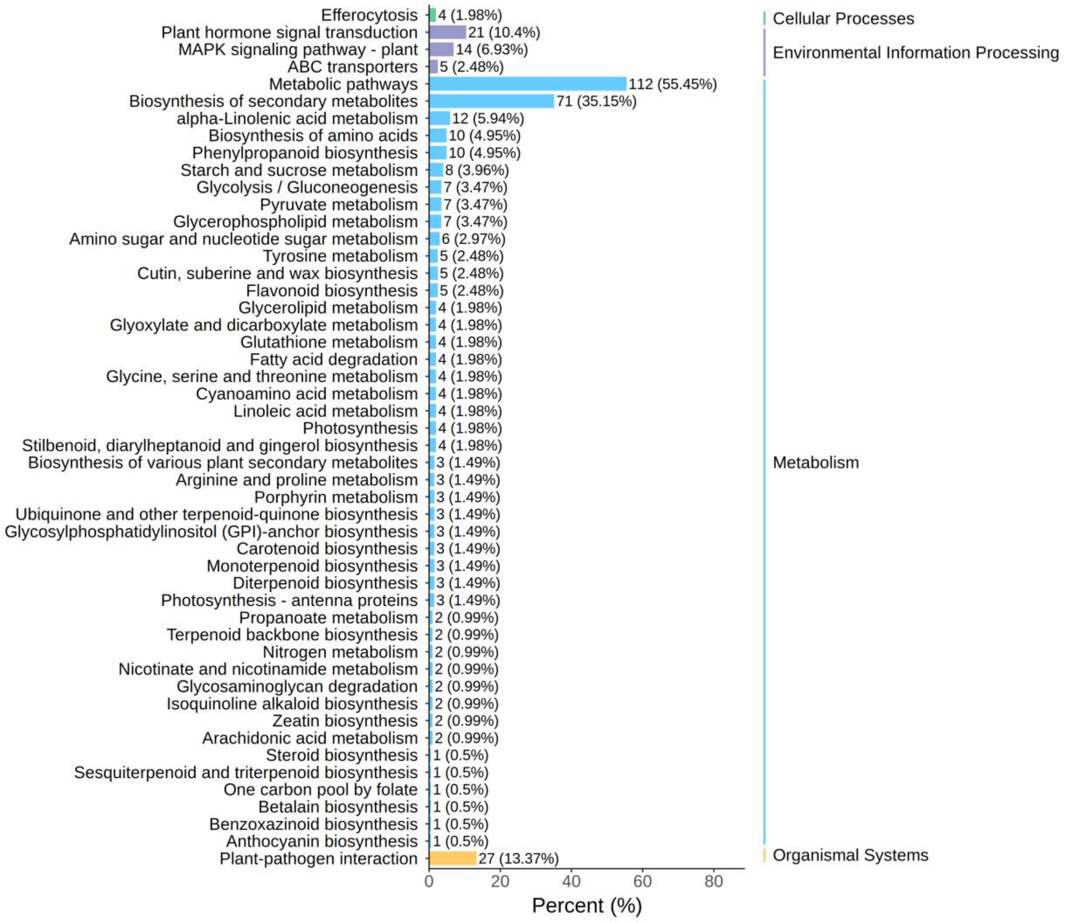

Additional Figure 4: KEGG enrichment analysis of 519 common DEGs in 6 comparative pairs. (Enrichment results were obtained from the Kyoto Encyclopedia o f Genes and Genomes database and is reproduced with permission, www.kegg.jp/kegg/kegg1.html)
