# Supplementary material for: Integrative metabolomic and transcriptomic analyses reveal flavonoid biosynthesis pathway in Eupatorium lindleyanum
Source: Sci Rep. 2025 Dec 4;15:43151. doi: 10.1038/s41598-025-27287-0 (PMC12678412; doi:10.1038/s41598-025-27287-0)
Supplement: Supplementary file 5 — Supplementary Material 5 [file 41598_2025_27287_MOESM5_ESM.pdf]

a

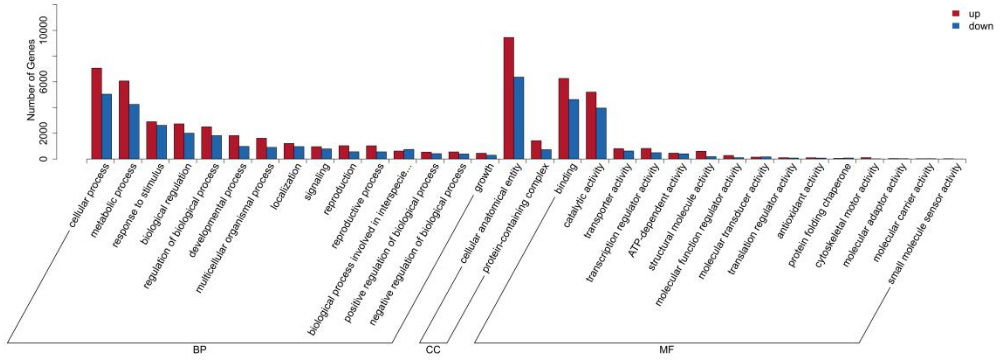

Number of Genes
0
2000
6000
10000
up
down
cellular process
metabolic process
response to stimulus
biological regulation
regulation of biological process
developmental process
multicellular organismal process
localization
signaling
reproduction
reproductive process
biological process involved in interspecie...
positive regulation of biological process
negative regulation of biological process
growth
cellular anatomical entity
protein-containing complex
binding
catalytic activity
transporter activity
transcription regulator activity
ATP-dependent activity
structural molecule activity
molecular function regulator activity
molecular transducer activity
translation regulator activity
antioxidant activity
protein folding chaperone
cytoskeletal motor activity
molecular adaptor activity
molecular carrier activity
small molecule sensor activity
BP
CC
MF

b

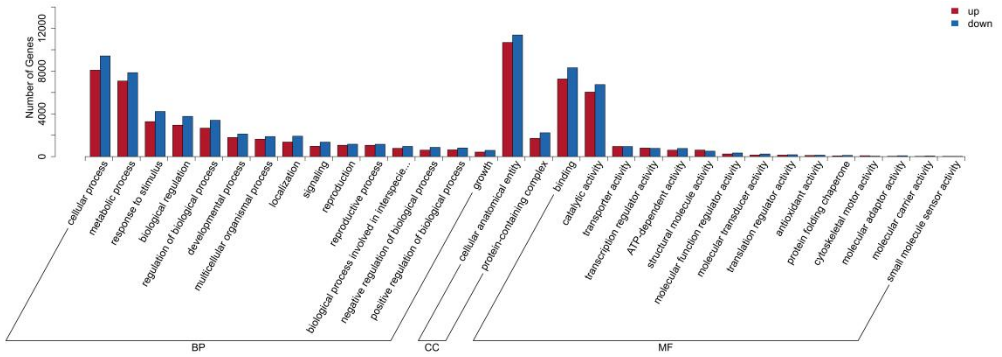

Number of Genes
0
4000
8000
12000
up
down
cellular process
metabolic process
response to stimulus
biological regulation
regulation of biological process
developmental process
multicellular organismal process
localization
signaling
reproduction
reproductive process
biological process involved in interspecie...
negative regulation of biological process
positive regulation of biological process
growth
cellular anatomical entity
protein-containing complex
binding
catalytic activity
transporter activity
transcription regulator activity
ATP-dependent activity
structural molecule activity
molecular function regulator activity
molecular transducer activity
translation regulator activity
antioxidant activity
protein folding chaperone
cytoskeletal motor activity
molecular adaptor activity
molecular carrier activity
small molecule sensor activity
BP
CC
MF

c

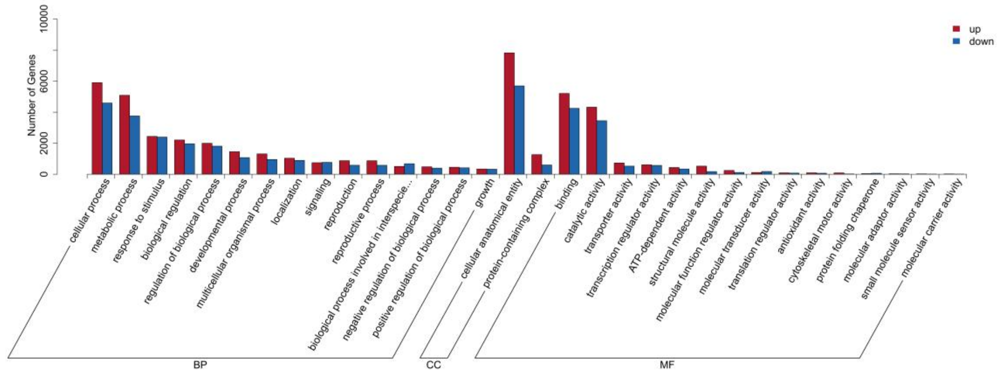

Number of Genes
0
2000
6000
10000
up
down
cellular process
metabolic process
response to stimulus
biological regulation
regulation of biological process
developmental process
multicellular organismal process
localization
signaling
reproduction
reproductive process
biological process involved in interspecie...
negative regulation of biological process
positive regulation of biological process
growth
cellular anatomical entity
protein-containing complex
binding
catalytic activity
transporter activity
transcription regulator activity
ATP-dependent activity
structural molecule activity
molecular function regulator activity
molecular transducer activity
translation regulator activity
antioxidant activity
cytoskeletal motor activity
protein folding chaperone
molecular adaptor activity
small molecule sensor activity
molecular carrier activity
BP
CC
MF

d

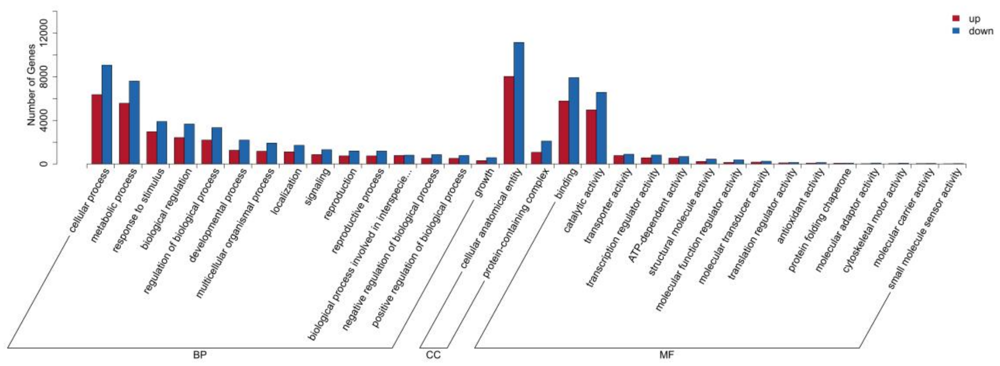

Number of Genes
0
4000
8000
12000
up
down
cellular process
metabolic process
response to stimulus
biological regulation
regulation of biological process
developmental process
multicellular organismal process
localization
signaling
reproduction
reproductive process
biological process involved in interspecie...
negative regulation of biological process
positive regulation of biological process
growth
cellular anatomical entity
protein-containing complex
binding
catalytic activity
transporter activity
transcription regulator activity
ATP-dependent activity
structural molecule activity
molecular function regulator activity
molecular transducer activity
translation regulator activity
antioxidant activity
protein folding chaperone
molecular adaptor activity
cytoskeletal motor activity
molecular carrier activity
small molecule sensor activity
BP
CC
MF

e

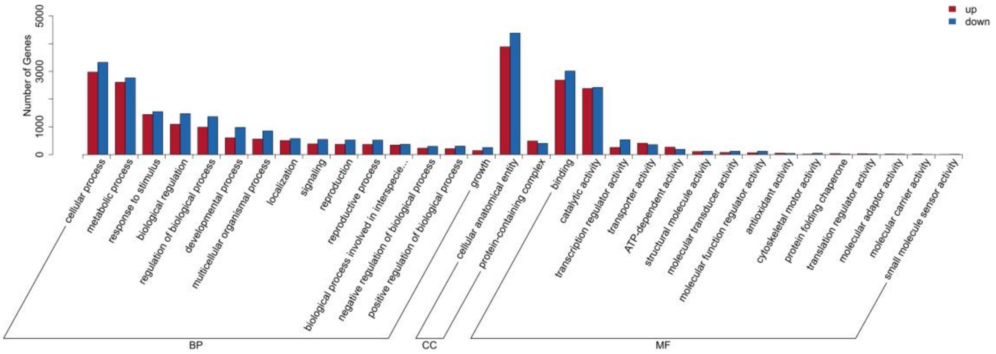

f

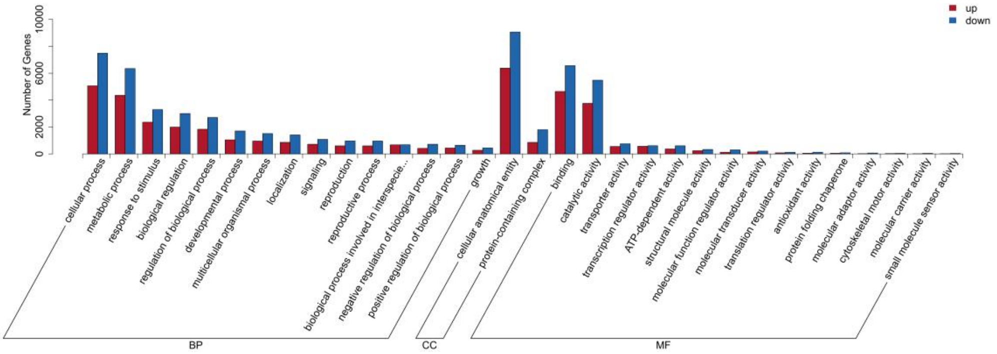

Additional Figure 5: Gene Ontology enrichment analyses of DEGs in *Eupatoriu m lindleyanum* across the six pairs of comparisons. (a: flower_vs_leaf; b: flowe r_vs_root; c: flower_vs_stem; d: leaf_vs_root; e: leaf_vs_stem; f: stem_vs_root)
