# Supplementary material for: Integrative metabolomic and transcriptomic analyses reveal flavonoid biosynthesis pathway in Eupatorium lindleyanum
Source: Sci Rep. 2025 Dec 4;15:43151. doi: 10.1038/s41598-025-27287-0 (PMC12678412; doi:10.1038/s41598-025-27287-0)
Supplement: Supplementary file 6 — Supplementary Material 6 [file 41598_2025_27287_MOESM6_ESM.pdf]

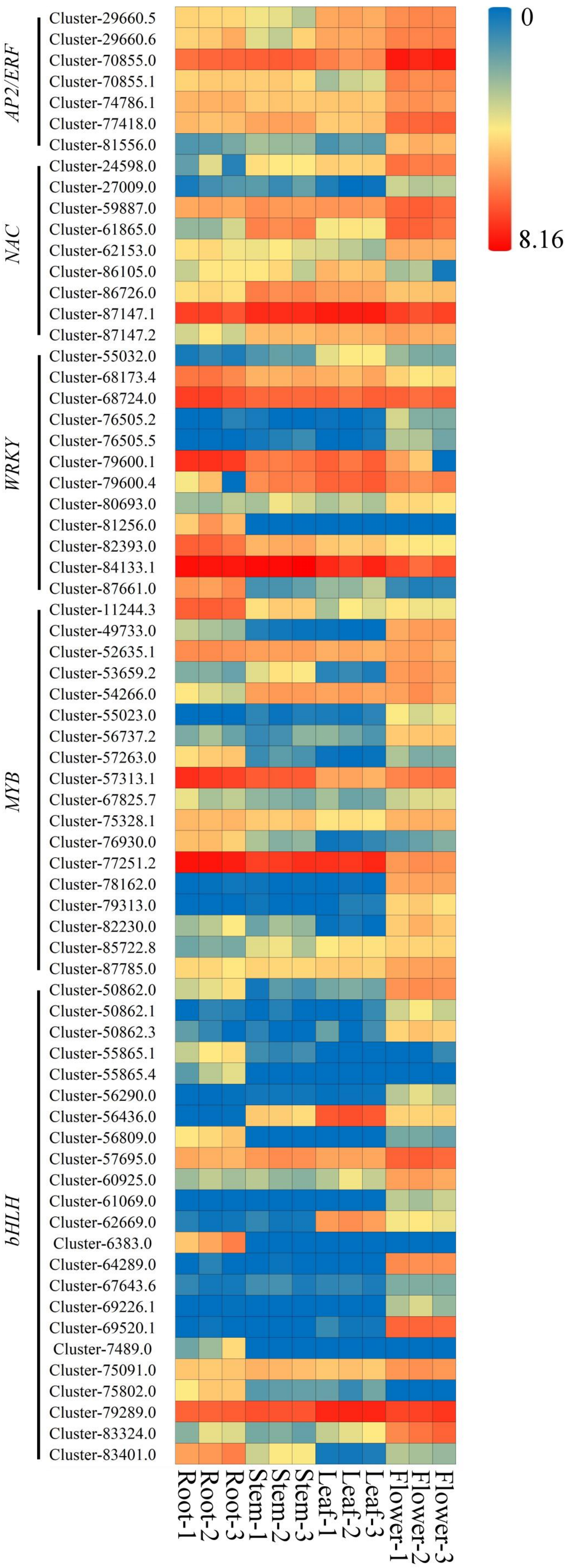
AP2/ERF
NAC
WRKY
MYB
bHLH
Cluster-29660.5
Cluster-29660.6
Cluster-70855.0
Cluster-70855.1
Cluster-74786.1
Cluster-77418.0
Cluster-81556.0
Cluster-24598.0
Cluster-27009.0
Cluster-59887.0
Cluster-61865.0
Cluster-62153.0
Cluster-86105.0
Cluster-86726.0
Cluster-87147.1
Cluster-87147.2
Cluster-55032.0
Cluster-68173.4
Cluster-68724.0
Cluster-76505.2
Cluster-76505.5
Cluster-79600.1
Cluster-79600.4
Cluster-80693.0
Cluster-81256.0
Cluster-82393.0
Cluster-84133.1
Cluster-87661.0
Cluster-11244.3
Cluster-49733.0
Cluster-52635.1
Cluster-53659.2
Cluster-54266.0
Cluster-55023.0
Cluster-56737.2
Cluster-57263.0
Cluster-57313.1
Cluster-67825.7
Cluster-75328.1
Cluster-76930.0
Cluster-77251.2
Cluster-78162.0
Cluster-79313.0
Cluster-82230.0
Cluster-85722.8
Cluster-87785.0
Cluster-50862.0
Cluster-50862.1
Cluster-50862.3
Cluster-55865.1
Cluster-55865.4
Cluster-56290.0
Cluster-56436.0
Cluster-56809.0
Cluster-57695.0
Cluster-60925.0
Cluster-61069.0
Cluster-62669.0
Cluster-6383.0
Cluster-64289.0
Cluster-67643.6
Cluster-69226.1
Cluster-69520.1
Cluster-7489.0
Cluster-75091.0
Cluster-75802.0
Cluster-79289.0
Cluster-83324.0
Cluster-83401.0
Root-1
Root-2
Root-3
Stem-1
Stem-2
Stem-3
Leaf-1
Leaf-2
Leaf-3
Flower-1
Flower-2
Flower-3
0
8.16

Additional Figure 6: The expression levels of flavonoid biosynthesis-related tran scription factors in roots, stems, leaves, and flowers.
